# Serum total cholesterol and low-density lipoprotein cholesterol are associated with Parkinson’s disease in East Asian populations: a bidirectional Mendelian randomization study with complementary animal observation

**DOI:** 10.3389/fnagi.2026.1841547

**Published:** 2026-07-22

**Authors:** Yanzi Bao, Huaxin Huang, Wei Feng, Fenglin Li, Qian Tang, Xi Li, Lingyue Liang, Jiang Lei, Yousheng Xiao

**Affiliations:** 1 Department of Neurology, The First Affiliated Hospital of Guangxi Medical University, Nanning, China; 2 Wuming Hospital Affiliated To Guangxi Medical University, Nanning, China; 3 Department of Neurology, Guangxi Hospital Division of the First Affiliated Hospital, Sun Yat-Sen University, Nanning, China

**Keywords:** colocalization, low-density lipoprotein cholesterol, Mendelian randomization, Parkinson’s disease, total cholesterol

## Abstract

**Background:**

Dysregulation of lipid metabolism has been implicated in Parkinson’s disease (PD), yet causal relationships remain unclear due to confounding factors inherent in observational studies. Most Mendelian randomization studies on lipid-PD relationships have been conducted in European populations, leaving the causal association in East Asian populations largely unexplored.

**Methods:**

We conducted a bidirectional Mendelian randomization (MR) analysis using genome-wide association study (GWAS) data from the Integrative Epidemiology Unit (IEU) Open GWAS project for East Asian populations to assess the causal associations between four lipid traits — triglycerides (TG), total cholesterol (TC), low-density lipoprotein cholesterol (LDL-C), and high-density lipoprotein cholesterol (HDL-C)—and PD. The primary MR analyses were conducted using the inverse-variance weighted (IVW) method. Sensitivity analyses included Cochran’s Q test, MR-Egger regression, and leave-one-out analyses. We further conducted colocalization analysis to assess whether the four lipid traits and PD shared common genetic variants. Finally, we complemented the findings using a 1-methyl-4-phenyl-1,2,3,6-tetrahydropyridine (MPTP)-induced mouse model of PD.

**Results:**

Genetically predicted higher levels of TC (OR = 0.476, 95% CI = 0.269–0.843, *p* = 0.011) and LDL-C (OR = 0.494, 95% CI = 0.289–0.845, *p* = 0.010) were associated with a reduced risk of PD. No causal relationship was observed between TG or HDL-C and PD. Reverse MR analysis indicated no causal effect of PD on any of the lipid traits. Colocalization analysis did not support shared genetic variants between the lipid traits and PD (all PP.H4 < 0.8). In MPTP-induced PD mice, both TC and LDL-C levels were significantly lower than in controls.

**Conclusion:**

This study provides genetic evidence supporting a genetically predicted inverse association of TC and LDL-C with PD risk among East Asian populations. The underlying mechanisms and any therapeutic implications warrant further experimental investigation.

## 1 Introduction

Parkinson’s disease (PD), the second most common progressive neurodegenerative disorder, is characterized by the loss of dopaminergic neurons in the substantia nigra of the midbrain and the pathological accumulation of *α*-synuclein in surviving neurons (Morris et al., 2024). Although PD has been known for over 200 years, its precise pathogenesis remains unclear. Advancing age is the strongest known risk factor for PD, with prevalence increasing exponentially after 60 years of age (Bloem et al., 2021). Currently, there is no cure for the condition. Clinical management primarily relies on a combination of levodopa, dopamine receptor agonists, and deep-brain stimulation to alleviate motor symptoms (Bloem et al., 2021; Hariz and Blomstedt, 2022). The onset and progression of PD are influenced by multiple factors, including environmental elements, genetic susceptibility, mitochondrial dysfunction, oxidative stress, neuroinflammation, and abnormal protein aggregation (Boyd et al., 2022; Liu et al., 2025). Growing evidence indicates that abnormalities in lipid metabolism contribute to PD pathogenesis through processes such as *α*-synuclein accumulation, mitochondrial and endoplasmic reticulum dysfunction, and ferroptosis (Tong et al., 2024; Galper et al., 2022). Therefore, drugs targeting lipid metabolism offer a promising approach to PD treatment (Li et al., 2024).

The causal link between lipid metabolism abnormalities and PD is still controversial. A meta-analysis suggested that metabolic syndrome is associated with increased risk of PD development in individuals with a strong genetic predisposition (Zhang et al., 2025). Similarly, a Finnish prospective study found that elevated total cholesterol (TC) levels are associated with increased PD risk (Hu et al., 2008). However, other studies have reported inconsistent findings. For instance, a systematic review suggested that lower levels of serum triglycerides (TG), high-density lipoprotein cholesterol (HDL-C), low-density lipoprotein cholesterol (LDL-C), and TC may be associated with reduced PD risk (Hong et al., 2022). Furthermore, large prospective studies have failed to identify a significant association between TC and PD (Liu et al., 2025; Simon et al., 2007). Observational studies in East Asian populations have reported lower serum TC and LDL-C levels in PD patients compared with healthy controls (Guo et al., 2015), and low LDL-C has been prospectively associated with increased PD risk (Huang et al., 2008). These inconsistencies likely arise from residual confounding and reverse causality in observational epidemiology, highlighting the need for more robust methods to study the causal link between lipid metabolism and PD.

Mendelian randomization (MR) uses genetic variation as an instrumental variable to mimic randomized controlled trials. This approach effectively reduces environmental confounding factors and reverse causality. It is widely used for causal inference in disease studies (Song Y. et al., 2025; Xue et al., 2025). In this study, bidirectional MR was performed using data from the Integrative Epidemiology Unit (IEU) Open GWAS project to investigate the causal relationship between blood lipids (TG, TC, LDL-C, and HDL-C) and PD in East Asian populations. Colocalization analysis was applied to further confirm the MR findings. Additionally, 1-methyl-4-phenyl-1,2,3,6-tetrahydropyridine (MPTP)-induced PD mouse models were used to determine serum TC and LDL-C levels. Ultimately, this study aims to investigate the causal association between blood lipids and PD using genetic approaches, providing a genetic rationale for future mechanistic studies.

## 2 Materials and methods

### 2.1 Study design

**Figure 1:**
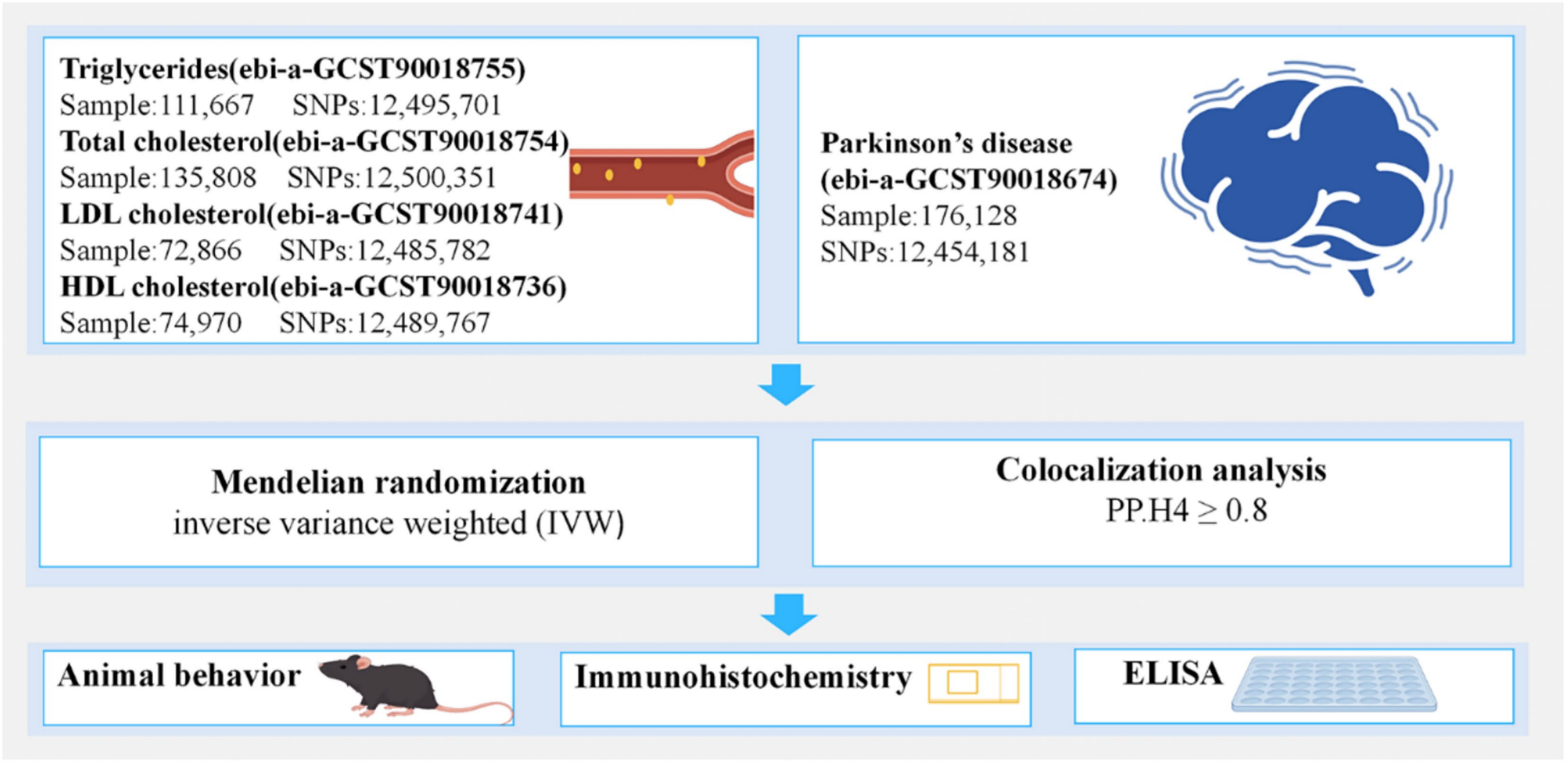
Flowchart showing the study design.

The study workflow is illustrated in Figure 1, using public summary data from the IEU Open GWAS project. All original GWAS included in the database obtained informed consent from participants and appropriate ethical approval. Since we only utilized de-identified, aggregate-level data, no additional ethical clearance was required for this analysis. First, we performed a bidirectional MR analysis to investigate the causal relationships between blood lipids (TG, TC, LDL-C, and HDL-C) and PD. Next, colocalization analysis was applied to corroborate the MR findings and to determine whether the observed associations were driven by shared causal genetic variants. This study adheres to the STROBE-MR guidelines for reporting Mendelian randomization studies. Finally, we measured changes in serum lipid levels using an MPTP-induced mouse model of PD for complementary animal observation.

### 2.2 Sources of lipid and PD data

All summary statistics were obtained from the IEU Open GWAS database.^1^ Details of the included GWAS are provided. Both the lipid traits, including TG, TC, LDL-C, and HDL-C, and the PD data were derived from East Asian populations.

### 2.3 MR analysis

We employed a rigorous MR framework to assess the causal effects between the lipid traits and PD. The instrument selection criteria were as follows: single nucleotide polymorphisms (SNPs) that were significantly associated with each exposure at a genome-wide significance threshold (*p* < 5 × 10^−8^) were identified. Clumping was performed to ensure SNP independence (window size 10,000 kb, r^2^ < 0.001). The F-statistic was then calculated, with a threshold of *F* > 10 required to exclude weak instrument bias (Song Y. et al., 2025; Xue et al., 2025). The MR analysis primarily employed inverse variance weighting (IVW) as the main method for causal effect estimation, with a significance threshold of *p* < 0.05. Effect sizes were expressed as odds ratios (OR) with 95% confidence intervals (CI). Sensitivity analyses included heterogeneity testing (Cochran’s Q test, *p* > 0.05 indicating no heterogeneity), pleiotropy testing (MR Egger intercept test, *p* > 0.05 suggesting no detectable pleiotropy), and a leave-one-out analysis to assess the robustness of causal estimates by sequentially excluding each SNP. Results were visualized using scatter plots, forest plots, and funnel plots. All analyses were performed using R software (version 4.2.3) and the ‘TwoSampleMR’ package (version 0.6.3). All instrumental SNPs were annotated using MyVariant.info, a variant annotation tool compatible with the ENSEMBL Variant Effect Predictor (VEP) pipeline. For each SNP, we retrieved overlapping or nearest genes, variant consequences (e.g., intergenic, intronic, non-coding transcript variant, missense), and regulatory region annotations.

### 2.4 Colocalization analysis

We performed colocalization analysis to evaluate whether the observed MR associations were driven by shared causal variants between the lipid trait and PD, as opposed to distinct causal variants in close linkage disequilibrium. This method tests five competing hypotheses: H0: the variant is associated with neither trait; H1: the variant is associated only with the lipid trait; H2: the variant is associated only with PD; H3: the variant is associated with both traits, but via two distinct causal variants; H4: the variant is associated with both traits via a single shared causal variant. Given the limited statistical power of colocalization analysis, we focused on the posterior probability for H4 (PP.H4). A PP.H4 value ≥ 0.8 was considered strong evidence for a shared causal variant at the locus (Song W. et al., 2025; Cui et al., 2023). All analyses were performed using R software (version 4.2.3).

### 2.5 MPTP-induced PD mouse model

All animal experiments were approved by the Animal Ethics Committee of Guangxi Medical University (approval no. 2024-S125-01) and were conducted in accordance with the Guide for the Care and Use of Laboratory Animals published by the National Research Council. Twelve male C57BL/6 mice, six weeks old and weighing 20–25 g, were purchased from the Animal Experiment Center of Guangxi Medical University. Male mice were chosen to avoid potential confounding effects of the estrous cycle on lipid metabolism and behavioral performance, which could introduce additional variability in this preliminary study. The mice were acclimatized for one week under standard conditions: temperature 21–22 °C; 12-h light/dark cycle (12 h light, 12 h dark); and ad libitum access to food and water. They were then randomly assigned to two groups (n = 6) using a random number table: the MPTP group and the control group. The MPTP group received intraperitoneal injections of MPTP hydrochloride solution (MCE, 23007-85-4) at a dose of 30 mg/kg per day for seven consecutive days. The control group received an equal volume of sterile 0.9% saline.

For subsequent behavioral tests and tissue collection, mice were anesthetized by intraperitoneal injection of 1.25% avertin at a dose of 0.2 mL per 10 g of body weight. The depth of anesthesia was confirmed by the absence of a withdrawal reflex to a toe pinch on the hind limb. Upon completion of all surgical procedures, mice that were under deep anesthesia were euthanized by cervical dislocation.

### 2.6 Behavioral testing

Behavioral tests were conducted sequentially on day 7 after the final injection. These tests were the open field test, the pole test, and the grasping test. Behavioral tests were recorded and analyzed by an investigator blinded to group allocation.

1. Open field test: Each mouse was placed in the center of a square open-field apparatus (90 cm × 90 cm × 40 cm). Using a video tracking system (SMART^®^ v3.0, Harvard Apparatus, United States), the total distance travelled and the time spent in the central area were recorded over a 5-min period.
2. Pole test: Each mouse was placed head-up at the top of a vertically rough wooden pole (55 cm long and 8 mm in diameter) wrapped with gauze to prevent slipping. The time taken for the mouse to climb to the base of the pole was recorded.
3. Grasping test: The mouse’s forelimbs were placed on a metal wire with a diameter of 1 mm (30 cm above the ground). The score was recorded over a 10-s period according to the following criteria: 0 points, falls; 1 point, forelimbs only hanging; 2 points, attempts to climb; 3 points, one hind limb grasps; 4 points, hangs with both hind limbs grasping the wire or escapes.

### 2.7 Immunohistochemistry

For immunohistochemical analysis, three mice per group were randomly selected. After transcranial perfusion with 4% paraformaldehyde to fix the tissue, the brains were harvested, paraffin-embedded, and sectioned into 5 μm-thick slices. Antigen retrieval was performed using EDTA (pH 9.0). Endogenous peroxidase was blocked using 3% H₂O₂, followed by blocking with 10% normal goat serum. The tyrosine hydroxylase (TH) primary antibody (Sigma, HY-15608, 1:200) was added, and the sections were incubated overnight at 4 °C. After washing with phosphate-buffered saline (PBS), a horseradish peroxidase (HRP)-conjugated secondary antibody was added, and the sections were incubated at room temperature for 20 min. Immunoreactivity was visualized using a 3,3′-diaminobenzidine (DAB) substrate kit according to the manufacturer’s instructions. Finally, the sections were counterstained with hematoxylin, dehydrated, cleared in xylene, and coverslipped with neutral resin. The TH-positive neurons in the substantia nigra pars compacta were observed and photographed under a light microscope. TH-positive neuron quantification: Automated counting was performed using ImageJ, with manual correction to ensure accuracy. The region of interest (ROI) was defined as the substantia nigra pars compacta (SNpc), delineated based on morphological landmarks. The counting area was standardized to a fixed size for each section.

### 2.8 Western blotting analysis

Total protein was extracted from the substantia nigra tissues of mice. The protein concentration was determined using a bicinchoninic acid (BCA) assay kit. Equal amounts of protein were separated by sodium dodecyl sulfate-polyacrylamide gel electrophoresis (SDS-PAGE) and subsequently transferred onto polyvinylidene fluoride (PVDF) membranes. The membranes were blocked with 5% non-fat milk in Tris-buffered saline with Tween 20 (TBST) for 1 h at room temperature and then incubated overnight at 4 °C with the following primary antibodies: TH (Sigma-Aldrich, HY-15608, 1:1000) and anti-glyceraldehyde-3-phosphate dehydrogenase (GAPDH) (Proteintech, 60004-1-Ig, 1:2000). After washing with TBST, the membranes were incubated with an HRP-conjugated secondary antibody for 1 h at room temperature. After further washing, the protein bands were visualized using an enhanced chemiluminescence kit according to the manufacturer’s instructions. The intensity of the bands was quantified using ImageJ software, with GAPDH serving as the loading control.

### 2.9 TC and LDL-C assays

Blood samples were collected from mice and centrifuged to obtain serum. Serum levels of TC and LDL-C were measured using a Mouse TC Assay Kit (Jiangsu Jinglai Bio, China, JL-T1371) and a Mouse LDL-C ELISA Kit (Jiangsu Jinglai Bio, China, JL18892), respectively, strictly following the manufacturer’s instructions.

### 2.10 Statistical analysis

Experimental data are expressed as mean ± SD. For animal experiments, between-group comparisons were performed using a two-tailed unpaired Student’s t-test for normally distributed data (assessed by the Shapiro–Wilk test), or the Mann–Whitney U test for non-normally distributed data. Statistical analysis was performed using GraphPad Prism 9.0 software; Differences were considered statistically significant when *p* < 0.05.

## 3 Results

### 3.1 SNPs used for MR analysis

The SNPs used in this study were selected based on the criteria described in the Methods section to ensure a strong association with the exposures and outcome (TG, TC, LDL-C, HDL-C, and PD). Ultimately, we identified 43, 49, 29, 50, and 6 independent instrumental SNPs for TG, TC, LDL-C, HDL-C, and PD, respectively, for the bidirectional MR analysis. The mean F-statistic for these SNPs was greater than 10, indicating that the instrumental variables were strong and that weak instrument bias in the MR analysis was unlikely. Variant annotation of the 177 instrumental SNPs revealed that most lipid-associated variants were located in non-coding regulatory regions, including introns, intergenic regions, and upstream/downstream regions. Notable examples include the missense variant APOE rs7412 (R176C, ε2 allele) specific to total cholesterol, and regulatory variants in LDLR, PCSK9, CELSR2/PSRC1, LIPC, and TOMM40. All six PD-associated instrumental variants were located in intergenic or non-coding regulatory regions. Detailed information for all SNPs, including annotation results, is listed in Supplementary Table 1.

### 3.2 Results of bidirectional Mendelian randomization analysis

**Figure 2:**
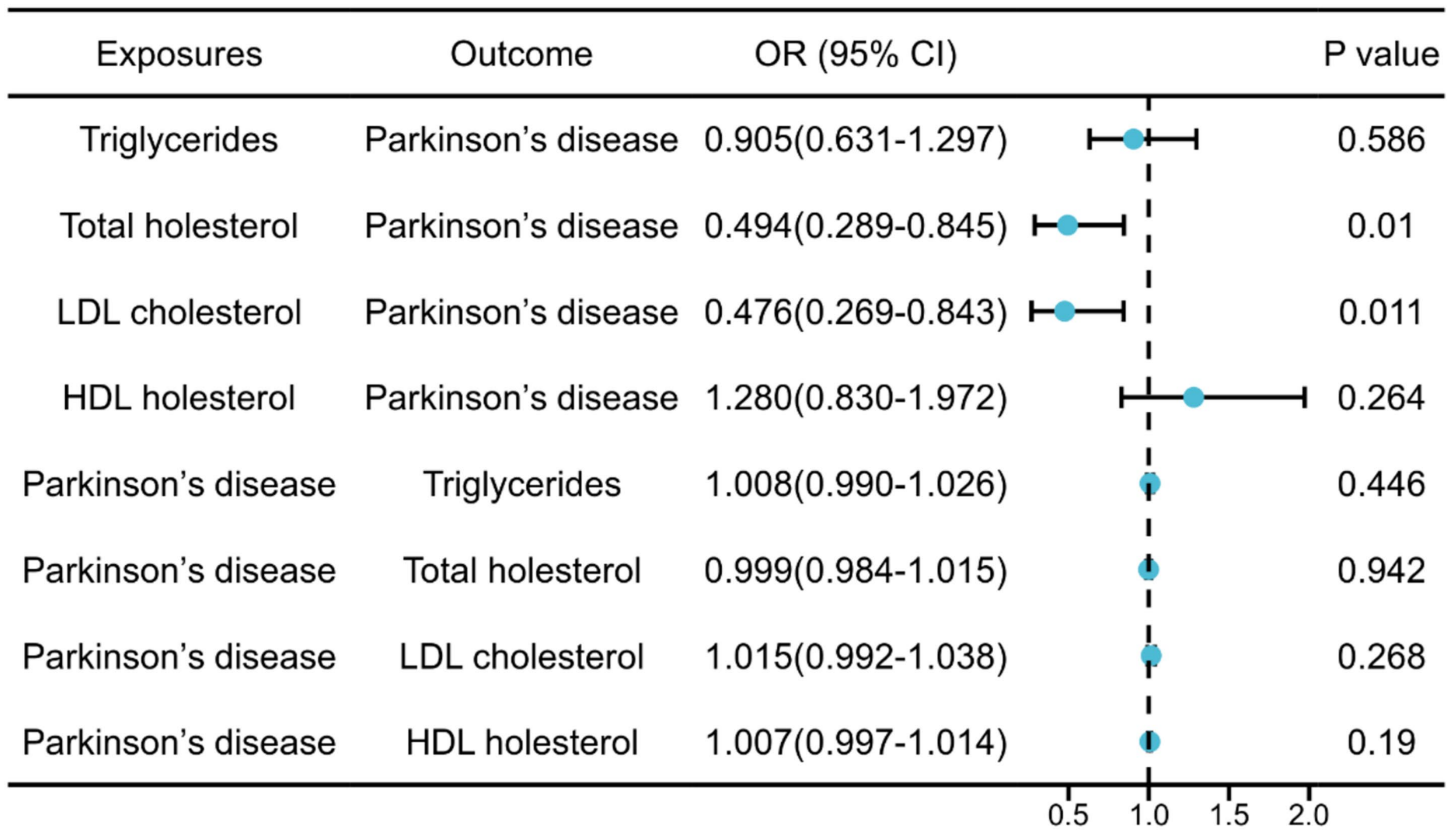
Bidirectional Mendelian randomization analysis of the association between blood lipids and Parkinson’s disease using the inverse-variance weighted method. LDL, Low-Density Lipoprotein; HDL, High-Density Lipoprotein; OR, Odds Ratio; 95%Cl, 95% Confidence Interval.

Forward MR analysis using the IVW method revealed that genetically predicted higher levels of TC (OR = 0.476, 95% CI = 0.269–0.843, *p* = 0.011) and LDL-C (OR = 0.494, 95% CI = 0.289–0.845, *p* = 0.010) were associated with a significantly reduced risk of PD. For each standard deviation increase in TC and LDL-C levels, the risk of developing PD decreased by approximately 52 and 51%, respectively. In contrast, TG (OR = 1.280, 95% CI = 0.830–1.972, *p* = 0.264) and HDL-C (OR = 0.905, 95% CI = 0.631–1.297, *p* = 0.586) showed no causal association with PD. Reverse MR analysis indicated no causal effect of PD on any of the four lipid traits (TG, TC, LDL-C, or HDL-C) (all *p* > 0.05), as summarized in Figure 2.

Sensitivity analyses revealed no significant heterogeneity in most analyses, except for the reverse MR analysis of PD on TC (Cochran’s Q statistic = 4.000, *p* = 0.025). All other analyses showed no evidence of heterogeneity (*p* > 0.05; Supplementary Table 2). Horizontal pleiotropy was assessed using the MR-Egger intercept method, which revealed no evidence of pleiotropy for any of the instrumental variables (all *p* > 0.05; Supplementary Table 3). Leave-one-out analysis confirmed the robustness of the MR results, demonstrating that the effect estimates were not driven by any single SNP (Supplementary Figure 1). Additional evidence from forest, scatter, and funnel plots supported the primary MR findings (Supplementary Figures 2–10).

### 3.3 Colocalization

To determine whether the observed associations between TC/LDL-C and PD shared the same causal genetic variants, we performed colocalization analysis. A posterior probability of PP.H4 ≥ 0.80 was considered strong evidence of colocalization. The analysis revealed no evidence of shared causal variants between the genetic instruments for PD and those for TC (PP.H4 = 0.065) or LDL-C (PP.H4 = 0.070), as the PP.H4 values were well below the 80% threshold. Regional colocalization plots for these analyses are presented in Supplementary Figure 11. Similarly, we found no evidence of colocalization for HDL-C or TG with PD (all PP.H4 < 0.8). The results of these analyses are visualized in Supplementary Figure 11. Detailed results of all colocalization analyses are provided in Supplementary Table 4.

### 3.4 MPTP-induced reduction of serum TC and LDL-C levels in mice

**Figure 3:**
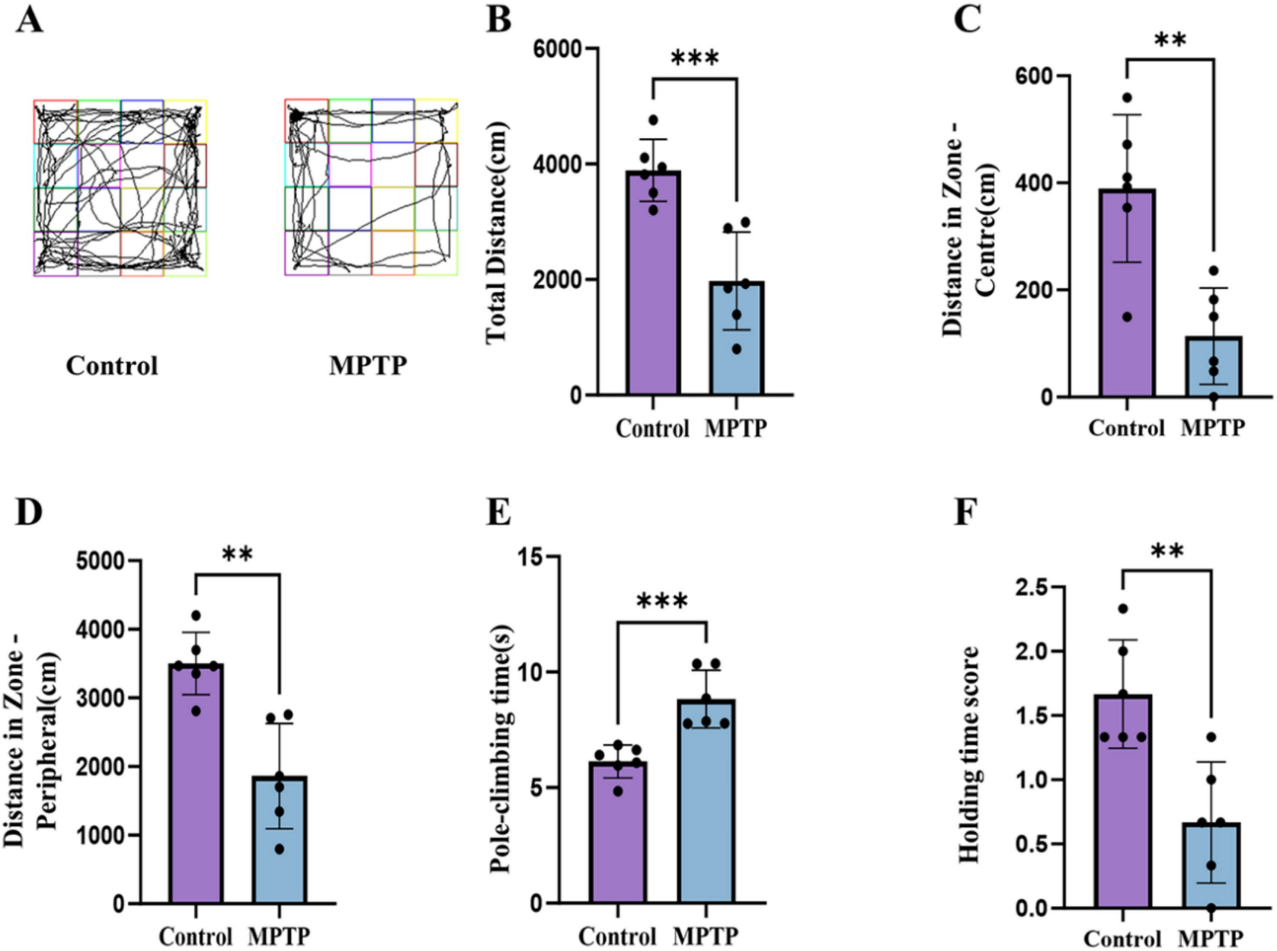
Behavioral assessments of MPTP-induced PD model mice **(A)**. Representative trajectories of mice in the open field test. **(B)** Total distance traveled **(C)**. Distance traveled in the central area **(D)**. Distance traveled in the peripheral area. **(E)** Descent time in the pole test **(F)**. Latency to fall in the grasping test. Data are shown as mean ± SD (*n* = 6 mice per group). Normality was assessed using the Shapiro–Wilk test; between-group comparisons were performed using a two-tailed unpaired Student’s t-test for normally distributed data (assessed by Shapiro–Wilk test), or Mann–Whitney U test for non-normally distributed data. Exact *p* values: **(B)** *p* < 0.001, **(C)** *p* = 0.002, **(D)** *p* = 0.001, **(E)** *p* < 0.001, **(F)** *p* = 0.003. *p < 0.05, **p < 0.01, ***p < 0.001 vs. control group.

**Figure 4:**
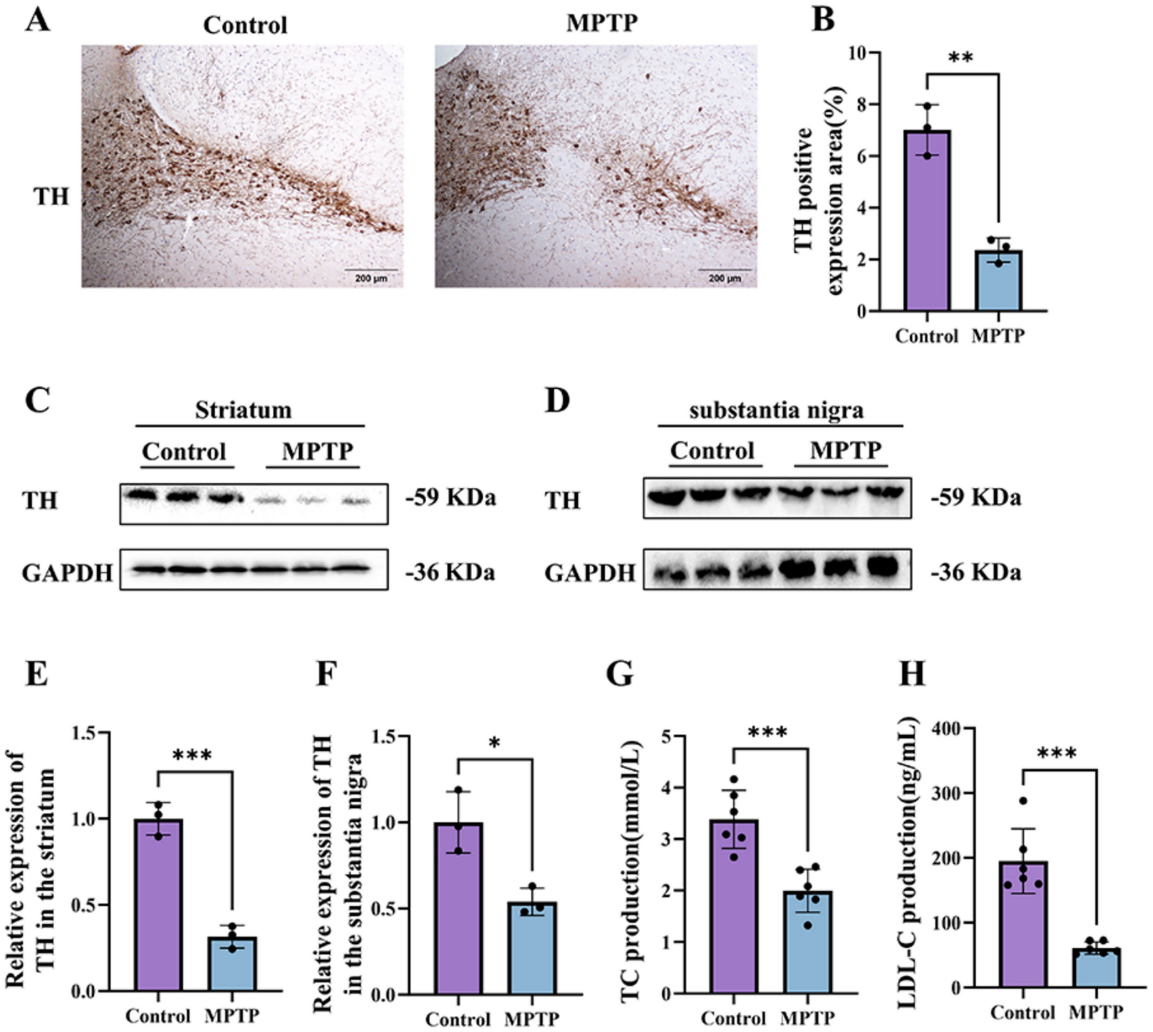
MPTP-induced dopaminergic neuron damage and serum lipid profile changes in mice. Representative images of TH-positive neurons in the substantia nigra pars compacta (SNpc). Scale bar, 500 μm **(A)**. Quantitative analysis of TH-positive expression area (%) **(B)**. Representative Western blot bands of TH protein in the substantia nigra (SN) and striatum (relative to GAPDH) **(C,D)**. Quantitative analysis of TH protein levels in the SN **(C)**. Quantitative analysis of TH protein levels in the striatum **(E,F)**. Serum lipid profile in MPTP-induced PD model mice. Serum LDL-C levels measured by ELISA **(G)**. Serum TC levels measured with a commercial assay kit **(H)**. For IHC and Western blot analyses **(A–F)**, *n* = 3 mice per group; for serum lipid measurements **(G,H)**, *n* = 6 mice per group. All data are shown as mean ± SD. Normality was assessed using the Shapiro–Wilk test; between-group comparisons were performed using a two-tailed unpaired Student’s t-test for normally distributed data (assessed by the Shapiro–Wilk test), or a Mann–Whitney U test for non-normally distributed data. Exact *p*-values: **(B)** *p* = 0.002, **(E)** *p* < 0.001, **(F)** *p* = 0.01, **(G)** *p* < 0.001, **(H)** *p* < 0.001. *p < 0.05, **p < 0.01, ***p < 0.001 vs. control group.

To complement our human genetic findings and to investigate whether PD-like pathology is associated with alterations in lipid profiles *in vivo*, we next employed an MPTP-induced mouse model of PD. To establish the MPTP-induced PD mouse model for complementary observational analysis, we administered MPTP for seven days. Afterwards, we assessed motor function and dopaminergic integrity. In the open field test, MPTP-treated mice traveled a significantly shorter total distance and explored less extensively than control mice (Figures 3A–D). In the pole test, the descent time was significantly longer for MPTP-treated mice than for controls (Figure 3E). Additionally, MPTP-treated mice showed a significantly shorter latency to fall in the Grasping test (Figure 3F). In line with the behavioral deficits, immunohistochemical and Western blot analyses revealed a significant reduction in both the number of TH-positive neurons and TH protein expression in MPTP-treated mice (Figures 4A–F). Collectively, these results suggest that our MPTP regimen induces motor impairment and dopaminergic neuron loss, consistent with previous reports using similar MPTP administration protocols in C57BL/6 mice (Song W. et al., 2025; Cui et al., 2023).

Given the association between genetically predicted lipid levels and PD risk identified in our MR analysis, we next sought to investigate the relationship between PD and lipid metabolism in our mouse model. We measured serum levels of LDL-C and TC in MPTP-treated and control mice using a Mouse LDL-C Assay Kit and a Mouse TC Assay Kit, respectively. The MPTP-treated mice exhibited a significant reduction in serum LDL-C levels compared to the control mice (Figure 4G). Moreover, serum TC levels were markedly decreased in the MPTP group compared to controls (Figure 4H). The MPTP-treated mice show reduced serum TC and LDL-C levels, indicating an association between PD-like pathology and altered lipid profiles.

## 4 Discussion

This study employed bidirectional MR to systematically investigate the causal relationship between blood lipids and PD in an East Asian population. The key finding was that genetically predicted higher levels of TC and LDL-C were associated with a significantly reduced risk of PD (TC: OR = 0.476, *p* = 0.011; LDL-C: OR = 0.494, *p* = 0.010). Conversely, no causal association was observed for TG or HDL-C. Reverse MR analysis ruled out reverse causality, indicating that PD does not influence blood lipid levels. Furthermore, MPTP-treated mice had significantly lower serum TC and LDL-C levels than control mice. These findings are not necessarily contradictory because each analysis tests a different causal direction. The reverse MR analysis found no significant evidence for genetics altering PD, then altering lipid levels. In contrast, the MPTP mouse experiment suggests that an induced PD-like neurotoxic state can reduce serum TC and LDL-C levels, independently of genetic state. Notably, the direct effect of lipid changes influencing PD pathobiology, suggested by the direct MR analysis, still requires further experimental validation. Collectively, our MR analysis supports a genetically predicted inverse association between TC/LDL-C and PD risk, while the MPTP mouse model shows that PD-like pathology is associated with reduced serum TC and LDL-C levels.

A key innovation of this study is its integrative approach, combining bidirectional MR, colocalization analyses, and complementary animal observation. This multi-tiered strategy effectively mitigated the confounding biases inherent in traditional observational studies, enabling more rigorous causal inference regarding the relationship between blood lipids and PD in East Asian populations. Specifically, the MR analysis employed genetic variants as instruments to mimic a randomized controlled trial, strengthening the reliability of causal estimates. Furthermore, the colocalization analysis ruled out the possibility that the observed associations were driven by genetic co-association, thereby further bolstering the credibility of our conclusions. Our findings help to resolve controversies from previous observational studies, which have reported inconsistent conclusions regarding the associations between the lipid traits (TG, TC, LDL-C, and HDL-C) and PD (Huang et al., 2008; Håglin et al., 2025; Miyake et al., 2010; Gudala et al., 2013). Notably, our results align with those of an MR study conducted in a European cohort, which also found that genetically predicted higher TC and LDL-C levels were associated with a reduced risk of PD (Fang et al., 2019). However, a notable discrepancy exists. While the European study suggested a protective effect of higher TG levels, we found no causal relationship between TG and PD in our East Asian population. This divergence highlights potential ethnic differences in lipid metabolism’s role in PD pathogenesis and underscores the importance of accounting for genetic and environmental backgrounds in future research.

Our animal studies showed that MPTP-treated mice had significantly lower serum TC and LDL-C levels than controls. This finding aligns with a clinical study of a Chinese population that reported significantly reduced TC and LDL-C levels in PD patients versus healthy individuals (Guo et al., 2015). We interpret these systemic lipid alterations as resulting mainly from PD-related metabolic dysregulation and impaired nutrient absorption, not from a direct protective or causative mechanism of the disease. However, the coexistence of reduced systemic cholesterol levels and neurodegenerative pathology highlights the necessity of distinguishing between systemic cholesterol levels and localized cholesterol metabolism within the brain. Intracellular cholesterol both promotes *α*-synuclein oligomerization and contributes to the formation of Lewy bodies (García-Sanz et al., 2021; Burré, 2015; Gegg et al., 2020). Furthermore, specific cholesterol metabolic enzymes and their products have been shown to accelerate the pathological aggregation and propagation of *α*-synuclein (Dai et al., 2025; Dai et al., 2023). Therefore, reduced systemic cholesterol levels may be a byproduct of the disease’s systemic manifestations rather than a protective adaptation. This distinction explains why statins, which inhibit peripheral cholesterol synthesis, exhibit neuroprotective potential. For instance, simvastatin has been demonstrated to safeguard neurons by mitigating α-Syn aggregation in both *in vitro* and *in vivo* settings by activating the PI3K/AKT survival pathway, inhibiting the caspase-3 apoptotic pathway, and exhibiting anti-inflammatory properties (Xu et al., 2013; Bar-On et al., 2008). These findings suggest that statins’ neuroprotective effects depend more on their ability to cross the blood–brain barrier and regulate the brain’s pro-pathogenic cholesterol microenvironment than on their capacity to lower systemic cholesterol levels. However, direct evidence linking our MR findings to statin action is lacking, and this requires dedicated experimental testing.

The relationship between statin use and Parkinson’s disease (PD) remains controversial. Preclinical studies have demonstrated pleiotropic effects of statins in PD models, including anti-inflammatory, antioxidant, and α-synuclein aggregation-reducing properties (Xu et al., 2013; Bar-On et al., 2008); however, observational epidemiological findings are discordant, with some studies reporting an association between statin use and reduced PD risk, whereas others find no association or even an increased risk (Wahner et al., 2008; Becker and Meier, 2009; Huang et al., 2011; Wu et al., 2022). Furthermore, two large-scale randomized controlled trials—the lovastatin trial in early PD patients and the PD STAT trial of simvastatin—failed to demonstrate that statins slow motor symptom progression in early PD (Stevens et al., 2022), and recent systematic reviews and meta-analyses have concluded that current clinical evidence is insufficient to support statins as a disease-modifying therapy for PD, warranting further high-quality studies (Gonçalves et al., 2025; Chiatto et al., 2026). Notably, the MR analysis in the present study revealed that genetically predicted higher TC/LDL-C levels are associated with a lower risk of PD. This genetic finding—an association between higher cholesterol and reduced PD risk—appears to contrast with the conventional expectation that statins confer benefits by lowering cholesterol. This apparent paradox may suggest that peripheral and cerebral cholesterol metabolism are governed by distinct pathways, and that the clinical effects of statins may be more closely related to their brain distribution and pleiotropic actions than to their systemic cholesterol-lowering effects.

The association between peripheral cholesterol levels and PD risk may reflect systemic metabolic alterations secondary to neurodegeneration, rather than a direct effect on neuronal membranes. Although the brain is the organ with the highest cholesterol concentration in the body, peripheral cholesterol and brain cholesterol are metabolically isolated from each other. Nevertheless, alterations in membrane cholesterol homeostasis do affect ion channel function—cholesterol depletion has been shown to almost completely suppress NMDA receptor currents and impair L-type calcium channel function (Korinek et al., 2015; Fortalezas et al., 2018). Furthermore, specific cholesterol metabolites such as 27-hydroxycholesterol can promote *α*-synuclein aggregation through pathways independent of peripheral cholesterol levels (Dai et al., 2023). Collectively, these observations underscore the complexity of the cholesterol-PD relationship and highlight the need for future studies to distinguish between peripheral and brain cholesterol metabolism, which holds both scientific and clinical value.

Colocalization analysis revealed no shared genetic variants (all PP.H4 < 0.8). The absence of colocalization (PP.H4 < 0.8) indicates that the MR associations are unlikely to be driven by shared causal variants at the tested loci. However, this finding should be interpreted cautiously, as it may also reflect that the genetic instruments for lipids and PD operate through distinct biological pathways, or that statistical power for colocalization was limited. Therefore, discordant MR and colocalization results do not necessarily weaken or strengthen the MR findings, but rather highlight the complexity of genetic pleiotropy.

Although the MR design has strengths, this study has several limitations. Firstly, the genetic data included only individuals of East Asian ancestry, which may limit the applicability of our findings to other ethnic groups. In addition, only male mice were used in the animal experiments, which limits the generalizability of our findings to females, given known sex differences in lipid metabolism and PD susceptibility. The small sample size for histological and molecular analyses (*n* = 3 per group for IHC and Western blot) limits statistical power and may affect the precision of inter-group variability estimates. Furthermore, ImageJ-based counting from selected sections is not equivalent to unbiased stereological analysis, and our findings from these preliminary experiments should be considered descriptive rather than mechanistically conclusive. Secondly, PD cases were not stratified into subgroups (e.g., hereditary versus idiopathic, or early- versus late-onset), so the observed causal estimates represent an average effect that may not apply to all PD subtypes. Thirdly, significant heterogeneity was detected in the analysis of PD in relation to TC, suggesting the presence of pleiotropic pathways or unmeasured confounders. We also note that while we performed separate MR analyses for each lipid as an independent exposure without adjusting for multiple comparisons—consistent with the hypothesis-driven nature of our study—we recognize that TC and LDL-C are biologically correlated, and this correlation should be considered when interpreting the findings. Finally, although our complementary animal observation provides parallel observational evidence that PD-like pathology is associated with reduced serum lipid levels, it remains preliminary and does not clarify how systemic lipid changes mechanistically influence brain pathology or vice versa. We explicitly acknowledge that the absence of cholesterol-lowering intervention experiments (e.g., statin treatment or genetic modulation of cholesterol metabolism) prevents us from experimentally validating the causal direction from lipids to PD that was suggested by the MR analysis. While our MR analysis supports a genetic-level causal association, the biological protective mechanism and its therapeutic potential remain open questions that require direct experimental investigation.

## Data Availability

Publicly available datasets were analyzed in this study. This data can be found at: https://opengwas.io/datasets/.

## Footnotes

1
https://opengwas.io/

## References

[ref1] Bar-On P. Crews L. Koob A. O. Mizuno H. Adame A. Spencer B. . (2008). Statins reduce neuronal alpha-synuclein aggregation in in vitro models of Parkinson’s disease. J. Neurochem. 105, 1656–1667. doi: 10.1111/j.1471-4159.2008.05254.x, 18248604 PMC2822545

[ref2] Becker C. Meier C. R. (2009). Statins and the risk of Parkinson disease: an update on the controversy. Expert Opin. Drug Saf. 8, 261–271. doi: 10.1517/14740330902859956, 19366342

[ref3] Bloem B. R. Okun M. S. Klein C. (2021). Parkinson’s disease. Lancet 397, 2284–2303. doi: 10.1016/S0140-6736(21)00218-X, 33848468

[ref4] Boyd R. J. Avramopoulos D. Jantzie L. L. McCallion A. S. (2022). Neuroinflammation represents a common theme amongst genetic and environmental risk factors for alzheimer and Parkinson diseases. J. Neuroinflammation 19:223. doi: 10.1186/s12974-022-02584-x, 36076238 PMC9452283

[ref5] Burré J. (2015). The synaptic function of α-synuclein. J. Parkinsons Dis. 5, 699–713. doi: 10.3233/Jpd-150642, 26407041 PMC4927875

[ref6] Chiatto L. M. Buccarello L. Carota G. Calabrò R. S. Rifici C. Esposito E. . (2026). The use of statins in Parkinson’s and alzheimer’s disease: a 2021-2025 state-of-the-art review of clinical and preclinical evidence. Pharmacol. Res. Perspect. 14:e70280. doi: 10.1002/prp2.70280, 42252551 PMC13243210

[ref7] Cui C. Song H. Han Y. Yu H. Li H. Yang Y. . (2023). Gut microbiota-associated taurine metabolism dysregulation in a mouse model of Parkinson’s disease. mSphere 8:e0043123. doi: 10.1128/msphere.00431-23, 37819112 PMC10732050

[ref8] Dai L. Wang J. Meng L. Zhang X. Xiao T. Deng M. . (2025). The cholesterol 24-hydroxylase Cyp46A1 promotes α-synuclein pathology in Parkinson’s disease. PLoS Biol. 23:e3002974. doi: 10.1371/journal.pbio.3002974, 39964974 PMC11835240

[ref9] Dai L. Wang J. Zhang X. Yan M. Zhou L. Zhang G. . (2023). 27-hydroxycholesterol drives the spread of α-synuclein pathology in Parkinson’s disease. Movem. Dis. 38, 2005–2018. doi: 10.1002/mds.29577, 37593929

[ref10] Fang F. Zhan Y. Hammar N. Shen X. Wirdefeldt K. Walldius G. . (2019). Lipids, apolipoproteins, and the risk of Parkinson disease. Circ. Res. 125, 643–652. doi: 10.1161/Circresaha.119.314929, 31382822

[ref11] Fortalezas S. Marques-Da-Silva D. Gutierrez-Merino C. (2018). Methyl-β-cyclodextrin impairs the phosphorylation of the β₂ subunit of L-type calcium channels and cytosolic calcium homeostasis in mature cerebellar granule neurons. Int. J. Mol. Sci. 19:3667. doi: 10.3390/ijms19113667, 30463327 PMC6275079

[ref12] Galper J. Dean N. J. Pickford R. Lewis S. J. G. Halliday G. M. Kim W. S. . (2022). Lipid pathway dysfunction is prevalent in patients with Parkinson’s disease. Brain 145, 3472–3487. doi: 10.1093/brain/awac176, 35551349 PMC9586542

[ref13] García-Sanz P. M F G A. J. Moratalla R. (2021). The role of cholesterol in α-synuclein and lewy body pathology in *Gba1* Parkinson’s disease. Mov. Disord. 36, 1070–1085. doi: 10.1002/mds.28396, 33219714 PMC8247417

[ref14] Gegg M. E. Verona G. Schapira A. H. V. (2020). Glucocerebrosidase deficiency promotes release of α-synuclein fibrils from cultured neurons. Hum. Mol. Genet. 29, 1716–1728. doi: 10.1093/hmg/ddaa085, 32391886 PMC7322566

[ref15] Gonçalves O. R. Nascimento A. B. Soares V. G. Abraham D. de Almeida Monteiro G. Freitas J. L. R. . (2025). Statins as potential disease-modifying therapy of Parkinson’s disease: a systematic review and meta-analysis with trial sequential analysis. Acta Neurol. Belg. 125, 915–922. doi: 10.1007/s13760-024-02709-4, 39738967

[ref16] Gudala K. Bansal D. Muthyala H. (2013). Role of serum cholesterol in Parkinson’s disease: a meta-analysis of evidence. J. Parkinsons Dis. 3, 363–370. doi: 10.3233/Jpd-130196, 23948990

[ref17] Guo X. Song W. Chen K. Chen X. P. Zheng Z. Cao B. . (2015). The serum lipid profile of Parkinson’s disease patients: a study from China. Int. J. Neurosci. 125, 838–844. doi: 10.3109/00207454.2014.979288, 25340257

[ref18] Håglin S. Edström M. Bäckman L. Håglin L. (2025). Investigating the impact of body weight changes and blood lipids on risk for mild cognitive impairment in Parkinson’s disease: a prospective cohort study. Am. J. Clin. Nutr. 122, 1086–1092. doi: 10.1016/j.ajcnut.2025.07.018, 40738204 PMC12674015

[ref19] Hariz M. Blomstedt P. (2022). Deep brain stimulation for Parkinson’s disease. J. Intern. Med. 292, 764–778. doi: 10.1111/joim.13541, 35798568 PMC9796446

[ref20] Hong X. Guo W. Li S. (2022). Lower blood lipid level is associated with the occurrence of Parkinson’s disease: a meta-analysis and systematic review. Int. J. Clin. Pract. 2022:9773038. doi: 10.1155/2022/9773038, 35801143 PMC9203242

[ref21] Hu G. Antikainen R. Jousilahti P. Kivipelto M. Tuomilehto J. (2008). Total cholesterol and the risk of Parkinson disease. Neurology 70, 1972–1979. doi: 10.1212/01.wnl.0000312511.62699.a8 18401018

[ref22] Huang X. Abbott R. D. Petrovitch H. Mailman R. B. Ross G. W. (2008). Low LDL cholesterol and increased risk of Parkinson’s disease: prospective results from Honolulu-Asia aging study. Mov. Disord. 23, 1013–1018. doi: 10.1002/mds.22013, 18381649

[ref23] Huang X. Auinger P. Eberly S. Oakes D. Schwarzschild M. Ascherio A. . (2011). Serum cholesterol and the progression of Parkinson’s disease: results from Datatop. PLoS One 6:e22854. doi: 10.1371/journal.pone.0022854, 21853051 PMC3154909

[ref24] Korinek M. Vyklicky V. Borovska J. Lichnerova K. Kaniakova M. Krausova B. . (2015). Cholesterol modulates open probability and desensitization of Nmda receptors. J. Physiol. 593, 2279–2293. doi: 10.1113/jphysiol.2014.288209, 25651798 PMC4457192

[ref25] Li H. Zeng F. Huang C. Pu Q. Thomas E. R. Chen Y. . (2024). The potential role of glucose metabolism, lipid metabolism, and amino acid metabolism in the treatment of Parkinson’s disease. CNS Neurosci. Ther. 30:e14411. doi: 10.1111/cns.14411, 37577934 PMC10848100

[ref26] Liu T. Kong X. Qiao J. Wei J. (2025). Decoding Parkinson’s disease: the interplay of cell death pathways, oxidative stress, and therapeutic innovations. Redox Biol. 85:103787. doi: 10.1016/j.redox.2025.103787, 40712453 PMC12312120

[ref27] Liu H. Peng T. Xu Y. Li Q. S. Yang L. F. Gong Z. . (2025). Association and biological pathways between metabolic syndrome and incident Parkinson’s disease: a prospective cohort study of 289,150 participants. Psychoneuroendocrinology 177:107444. doi: 10.1016/j.psyneuen.2025.107444, 40179596

[ref28] Miyake Y. Tanaka K. Fukushima W. Sasaki S. Kiyohara C. Tsuboi Y. . (2010). Case-control study of risk of Parkinson’s disease in relation to hypertension, hypercholesterolemia, and diabetes in Japan. J. Neurol. Sci. 293, 82–86. doi: 10.1016/j.jns.2010.03.002, 20347450

[ref29] Morris H. R. Spillantini M. G. Sue C. M. Williams-Gray C. H. (2024). The pathogenesis of Parkinson’s disease. Lancet 403, 293–304. doi: 10.1016/S0140-6736(23)01478-2, 38245249

[ref30] Simon K. C. Chen H. Schwarzschild M. Ascherio A. (2007). Hypertension, hypercholesterolemia, diabetes, and risk of Parkinson disease. Neurology 69, 1688–1695. doi: 10.1212/01.wnl.0000271883.45010.8a, 17761552 PMC2391077

[ref31] Song Y. Ye T. Roberts L. R. Larson N. B. Winham S. J. (2025). Mendelian randomization in hepatology: a review of principles, opportunities, and challenges. Hepatology 81, 1836–1846. doi: 10.1097/Hep.0000000000000649, 37874245

[ref32] Song W. Zhou Z. M. le Zhang L. Shu H.-f. Xia J.-r. Qin X. . (2025). Infiltrating peripheral monocyte Trem-1 mediates dopaminergic neuron injury in substantia nigra of Parkinson’s disease model mice. Cell Death Dis. 16:18. doi: 10.1038/s41419-025-07333-5, 39809747 PMC11733277

[ref33] Stevens K. N. Creanor S. Jeffery A. Whone A. Zajicek J. Foggo A. . (2022). Evaluation of simvastatin as a disease-modifying treatment for patients with Parkinson disease: a randomized clinical trial. JAMA Neurol. 79, 1232–1241. doi: 10.1001/jamaneurol.2022.3718, 36315128 PMC9623477

[ref34] Tong B. Ba Y. Li Z. Yang C. Su K. Qi H. . (2024). Targeting dysregulated lipid metabolism for the treatment of alzheimer’s disease and Parkinson’s disease: current advancements and future prospects. Neurobiol. Dis. 196:106505. doi: 10.1016/j.nbd.2024.106505, 38642715

[ref35] Wahner A. D. Bronstein J. M. Bordelon Y. M. Ritz B. (2008). Statin use and the risk of Parkinson disease. Neurology 70, 1418–1422. doi: 10.1212/01.wnl.0000286942.14552.51, 18184918 PMC3690297

[ref36] Wu C. C. Islam M. M. Lee A. J. Su C. H. Weng Y. C. Yeh C. Y. . (2022). Association between statin use and risk of Parkinson’s disease: evidence from 18 observational studies comprising 3.7 million individuals. J. Pers. Med. 12:825. doi: 10.3390/jpm12050825, 35629248 PMC9145914

[ref37] Xu Y. Q. Long L. Yan J. Q. Wei L. Pan M. Q. Gao H. M. . (2013). Simvastatin induces neuroprotection in 6-Ohda-lesioned Pc12 via the Pi3K/Akt/caspase 3 pathway and anti-inflammatory responses. CNS Neurosci. Ther. 19, 170–177. doi: 10.1111/cns.12053, 23279934 PMC6493537

[ref38] Xue C. C. Li H. Yu M. Chong C. C. Y. Fan Q. Tham Y. C. . (2025). Omega-3 fatty acids as protective factors for age-related macular degeneration: prospective cohort and mendelian randomization analyses. Ophthalmology 132, 598–609. doi: 10.1016/j.ophtha.2024.12.005, 39662686 PMC12126274

[ref39] Zhang X. Wang J. Dove A. Yu T. Li Q. Gottesman R. F. . (2025). Metabolic syndrome and incidence of Parkinson disease: a community-based longitudinal study and meta-analysis. Neurology 105:e214033. doi: 10.1212/Wnl.0000000000214033, 40834329 PMC12367420

