# Supplementary material for: Serum total cholesterol and low-density lipoprotein cholesterol are associated with Parkinson’s disease in East Asian populations: a bidirectional Mendelian randomization study with complementary animal observation
Source: Front Aging Neurosci. 2026 Jul 22;18:1841547. doi: 10.3389/fnagi.2026.1841547 (PMC13437702; doi:10.3389/fnagi.2026.1841547)
Supplement: Supplementary file 1 [file data_sheet_1.docx]

# Supplementary Table 1. Instrumental SNPs used for bidirectional Mendelian randomization analysis with variant annotation

| Exposure | SNP | Nearest Gene(s) | Consequence | | Effect Allele | | Other Allele | Beta | EAF | SE | P-value | Sample Size | R² | F-stat |
| --- | --- | --- | --- | --- | --- | --- | --- | --- | --- | --- | --- | --- | --- | --- |
| HDL cholesterol | rs10787431 | GPAM-TECTB | | intergenic_region | | G | A | -0.0319767 | 0.411865 | 0.00479414 | 2.60e-11 | 74970 | 0.000495369 | 37.1553 |
| HDL cholesterol | rs10899118 | DGAT2,LOC283214 | | intron_variant, upstream_gene_variant | | C | G | -0.0318561 | 0.349473 | 0.00494614 | 1.20e-10 | 74970 | 0.000461418 | 34.6075 |
| HDL cholesterol | rs11082764 | LIPG,LIPG-ACAA2 | | downstream_gene_variant, intergenic_region | | G | A | 0.0734488 | 0.461542 | 0.00471302 | 9.30e-55 | 74970 | 0.00268141 | 201.56 |
| HDL cholesterol | rs11199826 | WDR11-FGFR2 | | intergenic_region | | G | C | 0.0407504 | 0.194078 | 0.00608712 | 2.20e-11 | 74970 | 0.000519473 | 38.9641 |
| HDL cholesterol | rs11657865 | PGS1 | | intron_variant | | C | A | 0.0263838 | 0.433692 | 0.00476397 | 3.10e-08 | 74970 | 0.000341931 | 25.6427 |
| HDL cholesterol | rs117350179 | PPARG | | intron_variant | | G | C | -0.0307532 | 0.22931 | 0.00558318 | 3.60e-08 | 74970 | 0.000334282 | 25.0689 |
| HDL cholesterol | rs12293222 | LOC101929011-BUD13 | | intergenic_region | | A | G | -0.0659888 | 0.362248 | 0.00491692 | 4.60e-41 | 74970 | 0.002012 | 151.14 |
| HDL cholesterol | rs12446515 | HERPUD1-CETP | | intergenic_region | | T | C | 0.230404 | 0.210564 | 0.00580667 | 1.00e-200 | 74970 | 0.0176486 | 1346.85 |
| HDL cholesterol | rs12752223 | DR1-FNBP1L | | intergenic_region | | C | T | -0.0331974 | 0.614025 | 0.00484324 | 7.20e-12 | 74970 | 0.000522376 | 39.182 |
| HDL cholesterol | rs13306689 | MIR138-2-SLC12A3,SLC12A3 | | intergenic_region, upstream_gene_variant | | G | C | 0.141539 | 0.05211 | 0.010883 | 1.10e-38 | 74970 | 0.00197907 | 148.661 |
| HDL cholesterol | rs1358980 | VEGFA-LINC01512 | | intergenic_region | | T | C | -0.0307686 | 0.538888 | 0.00473147 | 7.90e-11 | 74970 | 0.00047049 | 35.2883 |
| HDL cholesterol | rs1532085 | AQP9-LIPC | | intergenic_region | | G | A | -0.10479 | 0.409372 | 0.00477521 | 9.80e-107 | 74970 | 0.00531009 | 400.212 |
| HDL cholesterol | rs1883025 | ABCA1 | | intron_variant | | T | C | -0.100492 | 0.278842 | 0.00521397 | 8.90e-83 | 74970 | 0.00406145 | 305.721 |
| HDL cholesterol | rs2070895 | AQP9-LIPC,LIPC,LIPC-AS1 | | downstream_gene_variant, intergenic_region, upstream_gene_variant | | A | G | 0.12626 | 0.515848 | 0.00470326 | 9.60e-159 | 74970 | 0.00796279 | 601.746 |
| HDL cholesterol | rs2274197 | ITPR3 | | intron_variant | | T | G | 0.0311724 | 0.235834 | 0.00558868 | 2.40e-08 | 74970 | 0.000350239 | 26.2659 |
| HDL cholesterol | rs2293440 | SCARB1 | | intron_variant | | G | A | 0.0277356 | 0.456864 | 0.00482999 | 9.30e-09 | 74970 | 0.000381769 | 28.6314 |
| HDL cholesterol | rs2297409 | ABCA1 | | intron_variant | | A | G | -0.0445906 | 0.376987 | 0.00483675 | 3.00e-20 | 74970 | 0.000933985 | 70.0845 |
| HDL cholesterol | rs255053 | DPEP2,DPEP3-DPEP2 | | downstream_gene_variant, intergenic_region | | A | G | 0.0657231 | 0.138583 | 0.00678697 | 3.50e-22 | 74970 | 0.00103131 | 77.3951 |
| HDL cholesterol | rs2925979 | CMIP | | intron_variant | | C | T | 0.0362758 | 0.662106 | 0.00495919 | 2.60e-13 | 74970 | 0.000588806 | 44.1676 |
| HDL cholesterol | rs2980859 | TRIB1-LINC00861 | | intergenic_region | | G | C | 0.0307801 | 0.680204 | 0.00503323 | 9.60e-10 | 74970 | 0.000412176 | 30.9127 |
| HDL cholesterol | rs328 | LPL | | stop_gained | | G | C | 0.141481 | 0.124304 | 0.00706881 | 4.10e-89 | 74970 | 0.00435777 | 328.123 |
| HDL cholesterol | rs34859606 | LPL-SLC18A1 | | intergenic_region | | G | C | 0.0878257 | 0.174873 | 0.0065916 | 1.70e-40 | 74970 | 0.00222596 | 167.248 |
| HDL cholesterol | rs3741521 | SCARB1 | | intron_variant | | A | G | -0.0455322 | 0.368171 | 0.00502603 | 1.30e-19 | 74970 | 0.000964531 | 72.3788 |
| HDL cholesterol | rs3759381 | CCDC63,CCDC63-MYL2,MYL2 | | downstream_gene_variant, intergenic_region | | T | G | 0.0380537 | 0.46205 | 0.00485063 | 4.30e-15 | 74970 | 0.000719871 | 54.0062 |
| HDL cholesterol | rs3803057 | OAS1 | | intron_variant | | A | G | -0.0435617 | 0.354825 | 0.0049058 | 6.70e-19 | 74970 | 0.000868823 | 65.1906 |
| HDL cholesterol | rs3808438 | TRPS1 | | intron_variant | | C | T | 0.0466016 | 0.769012 | 0.00558628 | 7.30e-17 | 74970 | 0.000771532 | 57.8849 |
| HDL cholesterol | rs4149310 | ABCA1 | | intron_variant | | T | A | 0.0583287 | 0.697312 | 0.00510529 | 3.10e-30 | 74970 | 0.00143621 | 107.824 |
| HDL cholesterol | rs4568938 | EXOC6 | | intron_variant | | T | G | 0.0320809 | 0.652812 | 0.00495208 | 9.30e-11 | 74970 | 0.000466526 | 34.9909 |
| HDL cholesterol | rs4766566 | CUX2 | | intron_variant | | T | C | 0.0848775 | 0.67556 | 0.00505827 | 3.40e-63 | 74970 | 0.00315801 | 237.5 |
| HDL cholesterol | rs4846913 | GALNT2 | | intron_variant | | A | C | 0.0341948 | 0.188598 | 0.00602295 | 1.40e-08 | 74970 | 0.000357868 | 26.8383 |
| HDL cholesterol | rs4917015 | C7orf72-IKZF1 | | intergenic_region | | T | C | 0.0283187 | 0.448457 | 0.00472703 | 2.10e-09 | 74970 | 0.000396713 | 29.7526 |
| HDL cholesterol | rs5094 | APOA4 | | intron_variant | | A | G | -0.0467681 | 0.102375 | 0.00782776 | 2.30e-09 | 74970 | 0.000401993 | 30.1487 |
| HDL cholesterol | rs55723461 | AQP9-LIPC | | intergenic_region | | G | T | 0.0526893 | 0.456381 | 0.0047138 | 5.20e-29 | 74970 | 0.00137752 | 103.412 |
| HDL cholesterol | rs59156491 | CEP164,CEP164-DSCAML1 | | downstream_gene_variant, intergenic_region | | T | C | 0.0336243 | 0.34453 | 0.00498147 | 1.50e-11 | 74970 | 0.000510642 | 38.3014 |
| HDL cholesterol | rs61941404 | CFAP73 | | intron_variant | | T | C | -0.0774932 | 0.149222 | 0.00714996 | 2.30e-27 | 74970 | 0.00152478 | 114.484 |
| HDL cholesterol | rs6488954 | SCARB1-UBC,UBC | | downstream_gene_variant, intergenic_region | | G | T | 0.0279224 | 0.578467 | 0.00476724 | 4.70e-09 | 74970 | 0.000380229 | 28.5159 |
| HDL cholesterol | rs6494006 | LIPC,LIPC-AS1 | | intron_variant | | C | T | -0.049983 | 0.217427 | 0.00570342 | 1.90e-18 | 74970 | 0.000850184 | 63.7908 |
| HDL cholesterol | rs651821 | APOA5,ZPR1 | | 5_prime_UTR_variant, upstream_gene_variant | | T | C | 0.142642 | 0.638729 | 0.00494954 | 1.20e-182 | 74970 | 0.00939019 | 710.637 |
| HDL cholesterol | rs67053123 | SCARB1-UBC | | intergenic_region | | A | T | 0.0688734 | 0.286081 | 0.00536693 | 1.10e-37 | 74970 | 0.00193763 | 145.542 |
| HDL cholesterol | rs6756501 | LDAH-APOB | | intergenic_region | | T | C | -0.0478864 | 0.080753 | 0.00860778 | 2.60e-08 | 74970 | 0.000340444 | 25.5311 |
| HDL cholesterol | rs7139170 | RPH3A | | intron_variant | | C | A | 0.0353122 | 0.498232 | 0.0047125 | 6.70e-14 | 74970 | 0.000623468 | 46.7693 |
| HDL cholesterol | rs7174514 | GCOM1-ALDH1A2 | | intergenic_region | | G | C | 0.0345089 | 0.228951 | 0.00597917 | 7.90e-09 | 74970 | 0.000420452 | 31.5337 |
| HDL cholesterol | rs72642490 | LIPG-ACAA2 | | intergenic_region | | T | C | 0.0436064 | 0.16127 | 0.00642481 | 1.10e-11 | 74970 | 0.000514406 | 38.5839 |
| HDL cholesterol | rs74613568 | CETP-NLRC5 | | intergenic_region | | A | G | 0.0656041 | 0.102835 | 0.00792106 | 1.20e-16 | 74970 | 0.000794155 | 59.5835 |
| HDL cholesterol | rs7778167 | MIR129-1,MIR129-1-LEP | | downstream_gene_variant, intergenic_region | | A | G | 0.0533474 | 0.110766 | 0.00746134 | 8.70e-13 | 74970 | 0.000560633 | 42.0531 |
| HDL cholesterol | rs78279385 | HLA-DRA-HLA-DRB5 | | intergenic_region | | A | G | 0.0379801 | 0.478006 | 0.00492554 | 1.20e-14 | 74970 | 0.000719848 | 54.0045 |
| HDL cholesterol | rs7938710 | CEP164-DSCAML1 | | intergenic_region | | T | C | -0.0406602 | 0.280287 | 0.0055075 | 1.60e-13 | 74970 | 0.000667008 | 50.0377 |
| HDL cholesterol | rs79616633 | NUP93 | | intron_variant | | T | C | 0.140965 | 0.050715 | 0.0107095 | 1.40e-39 | 74970 | 0.00191331 | 143.712 |
| HDL cholesterol | rs8059431 | CETP-NLRC5 | | intergenic_region | | T | C | -0.0955061 | 0.131337 | 0.00695685 | 6.90e-43 | 74970 | 0.00208128 | 156.355 |
| HDL cholesterol | rs940541 | GNAT3-CD36 | | intergenic_region | | G | A | -0.0408544 | 0.826263 | 0.00619619 | 4.30e-11 | 74970 | 0.000479202 | 35.942 |
| LDL cholesterol | rs11881156 | TMED1,TMED1-C19orf38 | | intergenic_region, upstream_gene_variant | | T | C | -0.0393331 | 0.167702 | 0.0063769 | 6.90e-10 | 72866 | 0.00043188 | 31.4821 |
| LDL cholesterol | rs12657266 | TIMD4-HAVCR1 | | intergenic_region | | T | C | 0.0378025 | 0.828884 | 0.00634354 | 2.50e-09 | 72866 | 0.000405374 | 29.5491 |
| LDL cholesterol | rs1277927 | SARS | | downstream_gene_variant, intron_variant | | T | G | 0.0912138 | 0.959657 | 0.0138046 | 3.90e-11 | 72866 | 0.000644222 | 46.9708 |
| LDL cholesterol | rs17111479 | BSND-PCSK9 | | intergenic_region | | C | G | -0.0706545 | 0.106739 | 0.00802907 | 1.40e-18 | 72866 | 0.000951943 | 69.4285 |
| LDL cholesterol | rs17145738 | BCL7B-TBL2,TBL2 | | downstream_gene_variant, intergenic_region | | T | C | 0.0447168 | 0.104328 | 0.00780944 | 1.00e-08 | 72866 | 0.000373698 | 27.2393 |
| LDL cholesterol | rs2179853 | MIR4422-PLPP3 | | intergenic_region | | C | A | -0.0413448 | 0.805223 | 0.00607195 | 9.80e-12 | 72866 | 0.000536199 | 39.0905 |
| LDL cholesterol | rs2376868 | RELB | | intron_variant | | A | C | 0.0360583 | 0.313782 | 0.00595927 | 1.40e-09 | 72866 | 0.000559926 | 40.8213 |
| LDL cholesterol | rs2738464 | LDLR | | 3_prime_UTR_variant | | C | G | 0.0770563 | 0.691906 | 0.00519144 | 7.70e-50 | 72866 | 0.00253149 | 184.923 |
| LDL cholesterol | rs3093679 | IFT20,TNFAIP1 | | intron_variant, upstream_gene_variant | | A | G | -0.0381919 | 0.156712 | 0.00656359 | 5.90e-09 | 72866 | 0.000385523 | 28.1016 |
| LDL cholesterol | rs3858704 | CUX2 | | intron_variant | | G | A | -0.0425826 | 0.674073 | 0.00515642 | 1.50e-16 | 72866 | 0.000796749 | 58.1006 |
| LDL cholesterol | rs41280378 | OIT3,PLA2G12B | | 3_prime_UTR_variant, downstream_gene_variant, non_coding_transcript_exon_variant | | G | T | -0.0454309 | 0.249555 | 0.00583527 | 6.90e-15 | 72866 | 0.000773068 | 56.3724 |
| LDL cholesterol | rs449647 | APOE,TOMM40,TOMM40-APOE | | downstream_gene_variant, intergenic_region, upstream_gene_variant | | T | A | 0.112532 | 0.042863 | 0.0134735 | 6.70e-17 | 72866 | 0.00103906 | 75.7885 |
| LDL cholesterol | rs4671437 | TMEM17-EHBP1 | | intergenic_region | | C | G | -0.0396183 | 0.138983 | 0.00698515 | 1.40e-08 | 72866 | 0.00037566 | 27.3824 |
| LDL cholesterol | rs553427 | LOC101927787-LINC01132 | | intergenic_region | | T | C | 0.0338519 | 0.710205 | 0.00527773 | 1.40e-10 | 72866 | 0.000471705 | 34.3866 |
| LDL cholesterol | rs600038 | ABO,ABO-SURF6 | | intergenic_region, upstream_gene_variant | | T | C | -0.0516786 | 0.721245 | 0.00534815 | 4.30e-22 | 72866 | 0.00107388 | 78.3315 |
| LDL cholesterol | rs6129772 | ZHX3 | | intron_variant | | T | C | 0.0313155 | 0.735859 | 0.00542534 | 7.80e-09 | 72866 | 0.000381223 | 27.788 |
| LDL cholesterol | rs61679753 | TOMM40 | | intron_variant | | A | T | -0.441535 | 0.047287 | 0.0114515 | 1.00e-200 | 72866 | 0.0175656 | 1302.79 |
| LDL cholesterol | rs61941404 | CFAP73 | | intron_variant | | T | C | 0.0468481 | 0.150046 | 0.0072658 | 1.10e-10 | 72866 | 0.000559801 | 40.8122 |
| LDL cholesterol | rs629301 | CELSR2,PSRC1 | | 3_prime_UTR_variant, downstream_gene_variant | | T | G | 0.107755 | 0.928101 | 0.00924168 | 2.00e-31 | 72866 | 0.00154961 | 113.086 |
| LDL cholesterol | rs6597617 | OBP2B-ABO | | intergenic_region | | G | C | 0.046522 | 0.254338 | 0.0055737 | 7.00e-17 | 72866 | 0.000820918 | 59.8645 |
| LDL cholesterol | rs737337 | ANGPTL8,DOCK6 | | synonymous_variant, upstream_gene_variant | | C | T | -0.0301973 | 0.265344 | 0.00542436 | 2.60e-08 | 72866 | 0.000355516 | 25.9136 |
| LDL cholesterol | rs7523141 | BSND-PCSK9 | | intergenic_region | | T | C | 0.0599023 | 0.717068 | 0.0054172 | 2.00e-28 | 72866 | 0.00145599 | 106.244 |
| LDL cholesterol | rs75346744 | TDRD15-LOC645949 | | intergenic_region | | G | A | 0.144804 | 0.065014 | 0.00968774 | 1.60e-50 | 72866 | 0.0025492 | 186.219 |
| LDL cholesterol | rs75509845 | LDAH-APOB | | intergenic_region | | A | G | -0.074639 | 0.118311 | 0.00739757 | 6.10e-24 | 72866 | 0.00116226 | 84.7852 |
| LDL cholesterol | rs76898656 | AQP9-LIPC | | intergenic_region | | A | G | 0.0338894 | 0.269555 | 0.00541114 | 3.80e-10 | 72866 | 0.000452265 | 32.9687 |
| LDL cholesterol | rs7703282 | ANKDD1B,POLK-ANKDD1B | | intergenic_region, upstream_gene_variant | | C | A | 0.0583634 | 0.552543 | 0.00492868 | 2.40e-32 | 72866 | 0.00168434 | 122.934 |
| LDL cholesterol | rs77303550 | DHODH-HP | | intergenic_region | | T | C | -0.0564843 | 0.208157 | 0.00600499 | 5.10e-21 | 72866 | 0.00105176 | 76.7159 |
| LDL cholesterol | rs7780562 | MIR148A-NFE2L3 | | intergenic_region | | C | A | -0.0411775 | 0.678565 | 0.0053104 | 8.90e-15 | 72866 | 0.000739664 | 53.9348 |
| LDL cholesterol | rs79106033 | LDAH-APOB | | intergenic_region | | T | C | 0.105353 | 0.11278 | 0.00833958 | 1.40e-36 | 72866 | 0.0022212 | 162.206 |
| Total cholesterol | rs112784971 | CYP7A1 | | intron_variant | | T | C | -0.0329446 | 0.299154 | 0.0038903 | 2.50e-17 | 135808 | 0.000455109 | 61.8347 |
| Total cholesterol | rs11748027 | ANKDD1B | | intron_variant | | T | C | 0.0485532 | 0.55474 | 0.0036716 | 6.40e-40 | 135808 | 0.00116458 | 158.341 |
| Total cholesterol | rs12024419 | DOCK7 | | intron_variant | | A | G | -0.0353863 | 0.104814 | 0.00584244 | 1.40e-09 | 135808 | 0.000234981 | 31.9193 |
| Total cholesterol | rs1260334 | GCKR,GCKR-C2orf16 | | downstream_gene_variant, intergenic_region | | A | C | -0.0350267 | 0.436295 | 0.00361153 | 3.10e-22 | 135808 | 0.000603477 | 82.0053 |
| Total cholesterol | rs12718462 | APOA1,APOA1-AS,APOC3,APOC3-APOA1 | | downstream_gene_variant, intergenic_region, upstream_gene_variant | | C | T | -0.0570656 | 0.077625 | 0.0067498 | 2.80e-17 | 135808 | 0.000466324 | 63.3592 |
| Total cholesterol | rs12720838 | APOB | | intron_variant | | T | C | -0.0624778 | 0.152351 | 0.00497048 | 3.10e-36 | 135808 | 0.00100819 | 137.057 |
| Total cholesterol | rs1277927 | SARS | | downstream_gene_variant, intron_variant | | T | G | 0.0581499 | 0.959551 | 0.0102342 | 1.30e-08 | 135808 | 0.000262485 | 35.6563 |
| Total cholesterol | rs13306501 | LDLR | | intron_variant | | A | G | 0.0351815 | 0.107678 | 0.00573842 | 8.70e-10 | 135808 | 0.000237852 | 32.3094 |
| Total cholesterol | rs16970987 | LINC01572 | | intron_variant | | C | G | -0.0257245 | 0.233904 | 0.00421903 | 1.10e-09 | 135808 | 0.000237162 | 32.2156 |
| Total cholesterol | rs1800588 | AQP9-LIPC,LIPC,LIPC-AS1 | | downstream_gene_variant, intergenic_region, upstream_gene_variant | | T | C | 0.0583891 | 0.507242 | 0.00356397 | 2.50e-60 | 135808 | 0.00170429 | 231.847 |
| Total cholesterol | rs2043082 | AQP9-LIPC | | intergenic_region | | A | G | 0.0427787 | 0.429195 | 0.0036165 | 2.80e-32 | 135808 | 0.00089666 | 121.881 |
| Total cholesterol | rs2278028 | DHODH | | intron_variant | | A | G | 0.0417622 | 0.113365 | 0.00573434 | 3.30e-13 | 135808 | 0.000350607 | 47.6312 |
| Total cholesterol | rs2297409 | ABCA1 | | intron_variant | | A | G | -0.0205902 | 0.377703 | 0.00366986 | 2.00e-08 | 135808 | 0.000199296 | 27.071 |
| Total cholesterol | rs2495505 | BSND-PCSK9 | | intergenic_region | | T | C | -0.0305704 | 0.369127 | 0.0037959 | 8.00e-16 | 135808 | 0.000435261 | 59.1368 |
| Total cholesterol | rs2575876 | ABCA1 | | intron_variant | | A | G | -0.0549584 | 0.272866 | 0.00398745 | 3.20e-43 | 135808 | 0.00119857 | 162.968 |
| Total cholesterol | rs2642438 | MARC1 | | missense_variant | | G | A | 0.0280596 | 0.818067 | 0.00465003 | 1.60e-09 | 135808 | 0.000234365 | 31.8357 |
| Total cholesterol | rs2738464 | LDLR | | 3_prime_UTR_variant | | C | G | 0.0590346 | 0.687669 | 0.00384263 | 2.90e-53 | 135808 | 0.00149706 | 203.614 |
| Total cholesterol | rs35279796 | RCN3-NOSIP | | intergenic_region | | A | G | -0.0369632 | 0.140118 | 0.00582246 | 2.20e-10 | 135808 | 0.000329232 | 44.7264 |
| Total cholesterol | rs360799 | EHBP1 | | intron_variant | | A | G | -0.0235823 | 0.22436 | 0.00426842 | 3.30e-08 | 135808 | 0.000193557 | 26.2912 |
| Total cholesterol | rs3808477 | TRPS1 | | intron_variant | | T | C | -0.0253181 | 0.268678 | 0.00401687 | 2.90e-10 | 135808 | 0.000251903 | 34.2185 |
| Total cholesterol | rs406315 | NECTIN2 | | downstream_gene_variant, intron_variant | | A | G | 0.164269 | 0.942771 | 0.0079183 | 1.30e-95 | 135808 | 0.00291181 | 396.597 |
| Total cholesterol | rs4072815 | LDAH-APOB | | intergenic_region | | T | G | -0.060163 | 0.117373 | 0.00551947 | 1.20e-27 | 135808 | 0.000749954 | 101.925 |
| Total cholesterol | rs4149310 | ABCA1 | | intron_variant | | T | A | 0.0411881 | 0.695853 | 0.00386561 | 1.70e-26 | 135808 | 0.000718083 | 97.59 |
| Total cholesterol | rs4573621 | GPAM-TECTB | | intergenic_region | | G | A | -0.0272205 | 0.274724 | 0.00398152 | 8.10e-12 | 135808 | 0.000295272 | 40.1115 |
| Total cholesterol | rs4794048 | KPNB1,KPNB1-TBKBP1 | | downstream_gene_variant, intergenic_region | | C | A | 0.0258034 | 0.352526 | 0.00374291 | 5.40e-12 | 135808 | 0.000303947 | 41.2903 |
| Total cholesterol | rs484084 | LINC01132,LOC101927787-LINC01132 | | intergenic_region, upstream_gene_variant | | C | T | 0.0267284 | 0.70586 | 0.00395848 | 1.50e-11 | 135808 | 0.000296653 | 40.2992 |
| Total cholesterol | rs55723461 | AQP9-LIPC | | intergenic_region | | G | T | 0.0220738 | 0.455915 | 0.00357781 | 6.80e-10 | 135808 | 0.000241732 | 32.8366 |
| Total cholesterol | rs55776147 | TIMD4-HAVCR1 | | intergenic_region | | G | A | 0.0413091 | 0.828003 | 0.00472719 | 2.40e-18 | 135808 | 0.000486042 | 66.0396 |
| Total cholesterol | rs55935382 | C7orf72-IKZF1 | | intergenic_region | | A | C | 0.0206269 | 0.43331 | 0.00360336 | 1.00e-08 | 135808 | 0.00020895 | 28.3826 |
| Total cholesterol | rs56156922 | HERPUD1-CETP | | intergenic_region | | C | T | 0.0540294 | 0.211557 | 0.00441107 | 1.70e-34 | 135808 | 0.000973841 | 132.382 |
| Total cholesterol | rs572512 | PCSK9 | | intron_variant | | T | C | 0.0400413 | 0.777562 | 0.00428573 | 9.40e-21 | 135808 | 0.000554613 | 75.3616 |
| Total cholesterol | rs57814779 | P4HA1 | | intron_variant | | A | C | -0.0233248 | 0.26236 | 0.00406946 | 9.90e-09 | 135808 | 0.000210576 | 28.6034 |
| Total cholesterol | rs600038 | ABO,ABO-SURF6 | | intergenic_region, upstream_gene_variant | | T | C | -0.0480602 | 0.723481 | 0.00397962 | 1.40e-33 | 135808 | 0.000924173 | 125.624 |
| Total cholesterol | rs602633 | CELSR2,CELSR2-PSRC1,PSRC1 | | downstream_gene_variant, intergenic_region | | G | T | 0.0752314 | 0.928625 | 0.00689457 | 1.00e-27 | 135808 | 0.000750265 | 101.967 |
| Total cholesterol | rs6129772 | ZHX3 | | intron_variant | | T | C | 0.0227844 | 0.733703 | 0.00402463 | 1.50e-08 | 135808 | 0.000202858 | 27.5549 |
| Total cholesterol | rs6597616 | OBP2B-ABO | | intergenic_region | | T | C | 0.0474736 | 0.253125 | 0.004146 | 2.30e-30 | 135808 | 0.000852152 | 115.826 |
| Total cholesterol | rs6663252 | USP24 | | intron_variant | | C | T | -0.057087 | 0.129241 | 0.00536 | 1.70e-26 | 135808 | 0.000733504 | 99.6874 |
| Total cholesterol | rs6689 | HLA-DQB1,HLA-DQB1-AS1 | | 3_prime_UTR_variant, non_coding_transcript_exon_variant | | G | A | 0.0291023 | 0.191232 | 0.00457171 | 1.90e-10 | 135808 | 0.000261981 | 35.5879 |
| Total cholesterol | rs67053123 | SCARB1-UBC | | intergenic_region | | A | T | 0.0285864 | 0.287466 | 0.00406629 | 2.10e-12 | 135808 | 0.000334766 | 45.4784 |
| Total cholesterol | rs6982502 | TRIB1-LINC00861 | | intergenic_region | | T | C | -0.0329372 | 0.564434 | 0.00358228 | 3.80e-20 | 135808 | 0.000533421 | 72.4805 |
| Total cholesterol | rs7188539 | ZFPM1 | | intron_variant | | C | T | -0.0215795 | 0.579581 | 0.00380623 | 1.40e-08 | 135808 | 0.000226939 | 30.8267 |
| Total cholesterol | rs73007599 | DNM2,TMED1 | | downstream_gene_variant, intron_variant, non_coding_transcript_exon_variant | | T | C | -0.032882 | 0.170925 | 0.00471644 | 3.10e-12 | 135808 | 0.00030644 | 41.6292 |
| Total cholesterol | rs73596816 | LPA | | intron_variant | | A | G | 0.0537796 | 0.041924 | 0.00955207 | 1.80e-08 | 135808 | 0.000232342 | 31.5608 |
| Total cholesterol | rs737337 | ANGPTL8,DOCK6 | | synonymous_variant, upstream_gene_variant | | C | T | -0.0477683 | 0.265879 | 0.004029 | 2.00e-32 | 135808 | 0.000890761 | 121.079 |
| Total cholesterol | rs7412 | APOE | | missense_variant | | T | C | -0.278855 | 0.041083 | 0.00913447 | 1.00e-200 | 135808 | 0.00612675 | 837.178 |
| Total cholesterol | rs75346744 | TDRD15-LOC645949 | | intergenic_region | | G | A | 0.0971366 | 0.062629 | 0.00733823 | 5.40e-40 | 135808 | 0.00110785 | 150.62 |
| Total cholesterol | rs77303550 | DHODH-HP | | intergenic_region | | T | C | -0.0583304 | 0.2098 | 0.00446156 | 4.60e-39 | 135808 | 0.00112814 | 153.381 |
| Total cholesterol | rs7780562 | MIR148A-NFE2L3 | | intergenic_region | | C | A | -0.0226848 | 0.678117 | 0.0039586 | 1.00e-08 | 135808 | 0.000224648 | 30.5154 |
| Total cholesterol | rs9958734 | LIPG | | 3_prime_UTR_variant | | C | T | 0.0426464 | 0.442732 | 0.00357841 | 9.60e-33 | 135808 | 0.000897428 | 121.986 |
| Triglycerides | rs1059611 | LPL | | 3_prime_UTR_variant | | C | T | -0.164316 | 0.125899 | 0.00600352 | 6.30e-165 | 111667 | 0.00594256 | 667.543 |
| Triglycerides | rs10822167 | JMJD1C | | intron_variant | | T | C | -0.0257609 | 0.696162 | 0.00436516 | 3.60e-09 | 111667 | 0.00028074 | 31.3576 |
| Triglycerides | rs112240819 | HLA-DRB1 | | intron_variant | | C | G | 0.0719652 | 0.101941 | 0.00765292 | 5.30e-21 | 111667 | 0.000948263 | 105.988 |
| Triglycerides | rs116234817 | HLA-B,HLA-C-HLA-B,MIR6891 | | downstream_gene_variant, intergenic_region | | A | G | 0.0662874 | 0.07239 | 0.00773251 | 1.00e-17 | 111667 | 0.000590114 | 65.934 |
| Triglycerides | rs12293222 | LOC101929011-BUD13 | | intergenic_region | | A | G | 0.0785054 | 0.358736 | 0.00418418 | 1.50e-78 | 111667 | 0.00283557 | 317.535 |
| Triglycerides | rs1260326 | GCKR | | missense_variant&splice_region_variant | | C | T | -0.0863985 | 0.442588 | 0.00403106 | 6.60e-102 | 111667 | 0.00368314 | 412.798 |
| Triglycerides | rs13103813 | DOK7 | | intron_variant | | C | A | -0.0242747 | 0.575298 | 0.00411037 | 3.50e-09 | 111667 | 0.000287949 | 32.163 |
| Triglycerides | rs1362076 | OR2H1-MAS1L | | intergenic_region | | T | G | 0.0615268 | 0.077596 | 0.0074795 | 1.90e-16 | 111667 | 0.0005419 | 60.5441 |
| Triglycerides | rs141943201 | HCG22 | | non_coding_transcript_exon_variant | | G | A | 0.067275 | 0.071541 | 0.00777053 | 4.80e-18 | 111667 | 0.00060125 | 67.179 |
| Triglycerides | rs1532085 | AQP9-LIPC | | intergenic_region | | G | A | -0.0425184 | 0.409022 | 0.00406611 | 1.40e-25 | 111667 | 0.000873981 | 97.6784 |
| Triglycerides | rs1800588 | AQP9-LIPC,LIPC,LIPC-AS1 | | downstream_gene_variant, intergenic_region, upstream_gene_variant | | T | C | 0.0643739 | 0.506968 | 0.00400361 | 3.60e-58 | 111667 | 0.0020716 | 231.805 |
| Triglycerides | rs1810322 | PLTP-PCIF1 | | intergenic_region | | A | G | 0.070955 | 0.059393 | 0.00850339 | 7.20e-17 | 111667 | 0.000562522 | 62.8494 |
| Triglycerides | rs2021163 | PPP1R11,RNF39 | | 3_prime_UTR_variant, downstream_gene_variant | | C | G | 0.0304028 | 0.162062 | 0.00543482 | 2.20e-08 | 111667 | 0.000251044 | 28.0399 |
| Triglycerides | rs2146324 | VEGFA,VEGFA-LINC01512 | | downstream_gene_variant, intergenic_region | | C | A | 0.0429186 | 0.885982 | 0.00654113 | 5.30e-11 | 111667 | 0.000372151 | 41.5717 |
| Triglycerides | rs2278426 | ANGPTL8,DOCK6 | | intron_variant, missense_variant, sequence_feature | | T | C | -0.0323162 | 0.264901 | 0.00453574 | 1.00e-12 | 111667 | 0.000406724 | 45.4353 |
| Triglycerides | rs2294212 | PLTP | | intron_variant, upstream_gene_variant | | G | C | -0.0272794 | 0.290339 | 0.00443399 | 7.60e-10 | 111667 | 0.000306659 | 34.2536 |
| Triglycerides | rs2803245 | ATG4C,ATG4C-LINC00466 | | downstream_gene_variant, intergenic_region | | T | C | 0.0289375 | 0.745645 | 0.00457907 | 2.60e-10 | 111667 | 0.000317632 | 35.4797 |
| Triglycerides | rs2954021 | TRIB1-LINC00861 | | intergenic_region | | G | A | -0.0602911 | 0.54765 | 0.00399577 | 1.90e-51 | 111667 | 0.001801 | 201.472 |
| Triglycerides | rs34859606 | LPL-SLC18A1 | | intergenic_region | | G | C | -0.109922 | 0.176447 | 0.00558769 | 3.70e-86 | 111667 | 0.0035116 | 393.505 |
| Triglycerides | rs35529421 | DOCK7 | | intron_variant | | A | T | -0.0702106 | 0.142379 | 0.00572554 | 1.40e-34 | 111667 | 0.00120386 | 134.591 |
| Triglycerides | rs369599 | NECTIN2 | | intron_variant | | T | C | -0.0276815 | 0.786994 | 0.00490034 | 1.60e-08 | 111667 | 0.000256905 | 28.6947 |
| Triglycerides | rs3775209 | AFF1 | | intron_variant | | A | G | 0.0312052 | 0.402335 | 0.00407501 | 1.90e-14 | 111667 | 0.000468306 | 52.3179 |
| Triglycerides | rs3782891 | MYL2 | | intron_variant | | A | G | 0.0277863 | 0.215281 | 0.00489388 | 1.40e-08 | 111667 | 0.000260862 | 29.1368 |
| Triglycerides | rs4389834 | MIR148A,NPVF-MIR148A | | downstream_gene_variant, intergenic_region | | A | G | 0.0229447 | 0.50326 | 0.00402853 | 1.20e-08 | 111667 | 0.000263218 | 29.4 |
| Triglycerides | rs4568938 | EXOC6 | | intron_variant | | T | G | -0.0348073 | 0.653882 | 0.00421658 | 1.50e-16 | 111667 | 0.000548396 | 61.2702 |
| Triglycerides | rs4665946 | ABHD1 | | intron_variant | | A | C | 0.0267103 | 0.315196 | 0.00431864 | 6.20e-10 | 111667 | 0.000307989 | 34.4021 |
| Triglycerides | rs4690098 | HGFAC | | intron_variant | | T | C | -0.0324384 | 0.418081 | 0.00404274 | 1.00e-15 | 111667 | 0.000512002 | 57.202 |
| Triglycerides | rs4846913 | GALNT2 | | intron_variant | | A | C | -0.0305379 | 0.188955 | 0.00510559 | 2.20e-09 | 111667 | 0.000285833 | 31.9266 |
| Triglycerides | rs4936358 | LOC101929011 | | intron_variant | | A | G | 0.0332899 | 0.444725 | 0.00412164 | 6.60e-16 | 111667 | 0.000547337 | 61.1518 |
| Triglycerides | rs55776147 | TIMD4-HAVCR1 | | intergenic_region | | G | A | 0.0299602 | 0.828453 | 0.00531349 | 1.70e-08 | 111667 | 0.000255135 | 28.4969 |
| Triglycerides | rs56156922 | HERPUD1-CETP | | intergenic_region | | C | T | -0.036558 | 0.212152 | 0.00495126 | 1.50e-13 | 111667 | 0.00044677 | 49.9109 |
| Triglycerides | rs58542926 | TM6SF2 | | stop_gained | | T | C | -0.058552 | 0.07873 | 0.00743846 | 3.50e-15 | 111667 | 0.000497325 | 55.5615 |
| Triglycerides | rs59156491 | CEP164,CEP164-DSCAML1 | | downstream_gene_variant, intergenic_region | | T | C | -0.0480907 | 0.344963 | 0.00422775 | 5.60e-30 | 111667 | 0.00104518 | 116.832 |
| Triglycerides | rs61863116 | CYP26A1,CYP26A1-MYOF | | downstream_gene_variant, intergenic_region | | G | A | 0.0277905 | 0.258369 | 0.00460227 | 1.60e-09 | 111667 | 0.000295972 | 33.0595 |
| Triglycerides | rs62392668 | SCGN,SCGN-HIST1H2AA | | downstream_gene_variant, intergenic_region | | A | C | 0.0509428 | 0.07933 | 0.00741055 | 6.20e-12 | 111667 | 0.000379085 | 42.3466 |
| Triglycerides | rs6494006 | LIPC,LIPC-AS1 | | intron_variant | | C | T | -0.032456 | 0.218636 | 0.00484091 | 2.00e-11 | 111667 | 0.000359911 | 40.2039 |
| Triglycerides | rs651821 | APOA5,ZPR1 | | 5_prime_UTR_variant, upstream_gene_variant | | T | C | -0.223665 | 0.644712 | 0.00422442 | 1.00e-200 | 111667 | 0.0229178 | 2619.14 |
| Triglycerides | rs6756501 | LDAH-APOB | | intergenic_region | | T | C | 0.0526792 | 0.080524 | 0.00732652 | 6.50e-13 | 111667 | 0.000410936 | 45.906 |
| Triglycerides | rs7417952 | DR1-FNBP1L | | intergenic_region | | G | C | 0.0257577 | 0.611619 | 0.00412681 | 4.30e-10 | 111667 | 0.000315198 | 35.2077 |
| Triglycerides | rs75627662 | APOC1,APOE,APOE-APOC1 | | downstream_gene_variant, intergenic_region, upstream_gene_variant | | T | C | 0.086891 | 0.120618 | 0.00626066 | 8.50e-44 | 111667 | 0.00160166 | 179.136 |
| Triglycerides | rs77048596 | MLXIPL | | intron_variant | | A | G | -0.10378 | 0.100137 | 0.00666983 | 1.40e-54 | 111667 | 0.00194101 | 217.165 |
| Triglycerides | rs7938710 | CEP164-DSCAML1 | | intergenic_region | | T | C | 0.0314148 | 0.280358 | 0.00466614 | 1.70e-11 | 111667 | 0.000398225 | 44.4855 |
| Triglycerides | rs9469053 | VARS | | intron_variant | | G | A | 0.0699506 | 0.075096 | 0.00759015 | 3.10e-20 | 111667 | 0.000679714 | 75.9519 |
| Parkinson's disease | rs11807932 | EIF2D | | intron_variant | | C | G | 1.50439 | 0.0210945 | 0.315001 | 1.79e-06 | 176128 | 0.0934675 | 18159.4 |
| Parkinson's disease | rs1433992 | RBMS3-TGFBR2 | | intergenic_region | | A | C | 0.909527 | 0.0567824 | 0.182944 | 6.64e-07 | 176128 | 0.0886108 | 17124 |
| Parkinson's disease | rs278948 | RBM47 | | intron_variant | | A | G | -0.372522 | 0.580105 | 0.0820616 | 5.64e-06 | 176128 | 0.0676055 | 12770.4 |
| Parkinson's disease | rs541615 | TEK | | intron_variant, sequence_feature | | G | A | -0.36093 | 0.441139 | 0.0780131 | 3.72e-06 | 176128 | 0.0642325 | 12089.6 |
| Parkinson's disease | rs646526 | KRT1,KRT2-KRT1 | | downstream_gene_variant, intergenic_region | | C | T | -0.462187 | 0.77046 | 0.0936205 | 7.94e-07 | 176128 | 0.0755569 | 14395.2 |
| Parkinson's disease | rs9375320 | NKAIN2 | | intron_variant | | A | G | 0.423791 | 0.211411 | 0.0958691 | 9.85e-06 | 176128 | 0.0598842 | 11219 |

**Note:** EAF = effect allele frequency; SE = standard error; R² = variance explained; F-stat = F-statistic for instrument strength.

**Key Findings:**
1. Only one missense variant identified: APOE rs7412 (R176C, ε2 allele), specific to Total cholesterol.
2. Most lipid-associated variants located in non-coding regulatory regions (introns, upstream/downstream, intergenic).
3. All six PD-associated instrumental variants were in intergenic or non-coding regulatory regions.
4. Notable genes: LDLR, APOE, PCSK9, CELSR2/PSRC1, LIPC, TOMM40, APOA1/APOC3.

**Supplementary Table 2. Heterogeneity tests**

| **id.exposure** | **id.outcome** | **method** | **Q** | **Q_df** | **Q_pval** |
| --- | --- | --- | --- | --- | --- |
| HDL cholesterol | Parkinson’s disease | MR Egger | 45.81 | 48 | 0.5632 |
| HDL cholesterol | Parkinson’s disease | Inverse variance weighted | 45.82 | 49 | 0.6029 |
| LDL cholesterol | Parkinson’s disease | MR Egger | 29.06 | 27 | 0.3579 |
| LDL cholesterol | Parkinson’s disease | Inverse variance weighted | 30.22 | 28 | 0.3529 |
| Total cholesterol levels | Parkinson’s disease | MR Egger | 36.86 | 47 | 0.8558 |
| Total cholesterol levels | Parkinson’s disease | Inverse variance weighted | 36.92 | 48 | 0.8775 |
| Triglycerides | Parkinson’s disease | MR Egger | 30.8 | 41 | 0.8771 |
| Triglycerides | Parkinson’s disease | Inverse variance weighted | 32.73 | 42 | 0.8469 |
| Parkinson’s disease | ebi-a-GCST90018736 | MR Egger | 0.7204 | 4 | 0.9488 |
| Parkinson’s disease | ebi-a-GCST90018736 | Inverse variance weighted | 1.549 | 5 | 0.9074 |
| Parkinson’s disease4 | ebi-a-GCST90018741 | MR Egger | 4.921 | 4 | 0.2955 |
| Parkinson’s disease | ebi-a-GCST90018741 | Inverse variance weighted | 5.515 | 5 | 0.3563 |
| Parkinson’s disease | ebi-a-GCST90018754 | MR Egger | 11.19 | 4 | **0.02451** |
| Parkinson’s disease | ebi-a-GCST90018754 | Inverse variance weighted | 13.61 | 5 | **0.01827** |
| Parkinson’s disease | ebi-a-GCST90018755 | MR Egger | 6.497 | 4 | 0.165 |
| Parkinson’s disease | ebi-a-GCST90018755 | Inverse variance weighted | 10.55 | 5 | 0.06104 |

**Supplementary Table 3. Pleiotropy tests**

| **id.exposure** | **id.outcome** | **egger_intercept** | **se** | **pval** |
| --- | --- | --- | --- | --- |
| HDL cholesterol | Parkinson’s disease | 0.00268176 | 0.0226856 | 0.906391 |
| LDL cholesterol | Parkinson’s disease | -0.0316791 | 0.030585 | 0.309495 |
| Total cholesterol | Parkinson’s disease | 0.00597581 | 0.026099 | 0.819888 |
| Triglycerides | Parkinson’s disease4 | -0.0325295 | 0.0234337 | 0.172589 |
| Parkinson’s disease | ebi-a-GCST90018736 | -0.00558134 | 0.00613231 | 0.414226 |
| Parkinson’s disease | ebi-a-GCST90018741 | -0.00482462 | 0.00694559 | 0.525523 |
| Parkinson’s disease | ebi-a-GCST90018754 | 0.00724575 | 0.00778658 | 0.404765 |
| Parkinson’s disease | ebi-a-GCST90018755 | 0.0105354 | 0.00666795 | 0.189256 |

**Supplementary Table 4. Colocalization analysis**

| **Phenotype1** | **Phenotype2** | **nsnps** | **PP.H0.abf** | **PP.H1.abf** | **PP.H2.abf** | **PP.H3.abf** | **PP.H4.abf** |
| --- | --- | --- | --- | --- | --- | --- | --- |
| HDL cholesterol | Parkinson’s disease | 658 | 4.25e-259 | 0.8924 | 2.59e-260 | 0.0543 | **0.05331** |
| LDL cholesterol | Parkinson’s disease | 356 | 3.00e-299 | 0.906 | 8.14e-301 | 0.02448 | **0.06952** |
| Total cholesterol | Parkinson’s disease | 379 | 4.10e-197 | 0.9101 | 1.11e-198 | 0.02468 | **0.0652** |
| Triglycerides | Parkinson’s disease | 555 | 2.36e-301 | 0.8598 | 1.35e-302 | 0.04887 | **0.09134** |

PP.H0 – posterior probability of no association with either trait; PP.H1 – posterior probability of association with lipid trait only; PP.H2 – posterior probability of association with PD only; PP.H3 – posterior probability of association with both traits via distinct causal variants; PP.H4 – posterior probability of association with both traits via a shared causal variant. PP.H4 values ≥ 0.80 were considered strong evidence of colocalization.
