# Supplementary material for: Serum total cholesterol and low-density lipoprotein cholesterol are associated with Parkinson’s disease in East Asian populations: a bidirectional Mendelian randomization study with complementary animal observation
Source: Front Aging Neurosci. 2026 Jul 22;18:1841547. doi: 10.3389/fnagi.2026.1841547 (PMC13437702; doi:10.3389/fnagi.2026.1841547)
Supplement: Supplementary file 2 [file data_sheet_2.docx]

Supplementary Figure 1. Leave-one-out sensitivity analysis: HDL -C on PD (A), LDL-C on PD (B), TC on PD (C), TG on PD(D).

Supplementary Figure 2. Leave-one-out sensitivity analysis: PD on HDL-C (A), PD on LDL-C (B), PD on TC (C), PD on TG (D).

Supplementary Figure 3. Forest plot(A), Scatter plot(B) and Funnel plot (C) of the association of HDL-C on PD.

Supplementary Figure 4. Forest plot(A), Scatter plot(B) and Funnel plot (C) of the association of LDL-C on PD.

Supplementary Figure 5. Forest plot(A), Scatter plot(B) and Funnel plot (C) of the association of TC on PD.

Supplementary Figure 6. Forest plot(A), Scatter plot(B) and Funnel plot (C) of the association of TG on PD.

Supplementary Figure 7. Forest plot(A), Scatter plot(B) and Funnel plot (C) of the association of PD on HDL-C.

Supplementary Figure 8. Forest plot(A), Scatter plot(B) and Funnel plot (C) of the association of PD on LDL-C.

Supplementary Figure 9. Forest plot(A), Scatter plot(B) and Funnel plot (C) of the association of PD on TC.

Supplementary Figure 10. Forest plot(A), Scatter plot(B) and Funnel plot (C) of the association of PD on TG.

Supplementary Figure 11. Colocalization analysis of blood lipids (TG and HDL-C) and PD. Regional colocalization plot for TG and PD(A). Regional colocalization plot for HDL-C and PD(B).
